# Purkinje cell-specific loss of Neurofascin and Ankyrin G causes disruption of axon initial segments, neurodegeneration, and cerebellar ataxia

**DOI:** 10.3389/fncel.2026.1690466

**Published:** 2026-04-13

**Authors:** Qian Shi, Anna M. Taylor, Lacey B. Sell, Manzoor A. Bhat

**Affiliations:** 1 Department of Cellular and Integrative Physiology, Center for Biomedical Neuroscience, Long School of Medicine, University of Texas Health Science Center, San Antonio, TX, United States; 2 Department of Neuroscience, University of Texas at Dallas, Richardson, TX, United States

**Keywords:** Ankyrin G, axon initial segment, cerebellum, neurodegeneration, Neurofascin 186, pinceau organization, Purkinje cells

## Abstract

The axon initial segment (AIS) is essential for initiating action potentials and maintaining neuronal polarity, yet the developmental roles of its core molecular components—Neurofascin 186 (NF186) and Ankyrin G (AnkG)—remain incompletely defined in cerebellar Purkinje cells. Here, we generated Purkinje cell-specific NF186 and AnkG single- and double-knockout mice to investigate how these adhesion and scaffolding proteins cooperatively regulate AIS formation, ion channel localization, synaptic targeting, and neuronal survival. We found that genetic ablation of either *Nfasc NF186* (*NFKO*) or *Ankyrin3* (*AnkGKO*) disrupted assembly and maintenance of the AIS cytoskeleton, and that this defect was exacerbated by combined loss of both proteins during postnatal development. Other AIS-enriched proteins, including βIV Spectrin (βIVSpec), voltage-gated sodium (Na_v_), and potassium (K_v_1.2) channels, failed to properly localize to the AIS and progressively disintegrated between postnatal days 10 and 30. Notably, K_v_1.2 clustering at the pinceau synapse was disrupted, and basket cell axons showed misaligned terminal organization, indicating defective inhibitory synapse innervation. By 2 months of age, degeneration of Purkinje cells was evident, accompanied by cerebellar dysfunction. Notably, *AnkG* ablation caused a progressive postnatal loss of NF186 at the AIS, whereas *NF* ablation resulted in much slower loss of AnkG at the AIS in Purkinje cells and closely phenocopied the severe AIS destabilization observed in *NF/AnkG* double-knockout mice. In addition, our RNA-seq analysis revealed that Purkinje cell-specific loss of NF186 predominantly activated immune-inflammatory pathways; AnkG loss significantly disrupted neuronal developmental and metabolic processes; and the dual loss of NF186/AnkG produced transcriptional changes that were distinct from, and in part intermediate to, those observed in NF186 and AnkG single knockout. Collectively, our results show that NF186 and AnkG have complementary, non-redundant roles in establishing and maintaining the Purkinje cell AIS, and that their loss disrupts synaptic organization at the AIS. These findings advance our understanding of AIS development in cerebellar neurons and have implications for diseases involving AIS dysfunction, including cerebellar ataxia and demyelinating neuropathies.

## Introduction

Neurons function as highly specialized units within neural circuits, integrating synaptic inputs and generating precise outputs for neuronal transmission. The ability of neurons to initiate and propagate action potentials is essential for circuit function and is largely determined by the axon initial segment (AIS), a specialized subcellular domain that serves as the site of action potential initiation and maintains axonal identity. The molecular composition and structural integrity of the AIS are essential for ensuring proper neuronal excitability and circuit stability (Freal and Hoogenraad, 2025). Disruptions in AIS organization are increasingly recognized as a contributing factor in neurodevelopmental disorders, neurodegeneration, and ataxias (Huang and Rasband, 2018). In the cerebellar microcircuit, Purkinje neurons play a central role as the sole output of the cerebellar cortex, integrating excitatory and inhibitory synaptic inputs to control motor coordination and learning. As large GABAergic projection neurons, Purkinje cells receive excitatory inputs from climbing and parallel fibers and inhibitory inputs from molecular layer interneurons, including basket cells, which form the pinceau structure around the Purkinje AIS (Sakaba, 2008). The highly specialized AIS of Purkinje neurons ensures high-fidelity spike generation, which is crucial for precise motor activity and execution. The structural and functional integrity of the Purkinje AIS is, therefore, essential for maintaining normal cerebellar function.

The AIS is defined by a highly ordered molecular assembly, anchored by the scaffold protein Ankyrin G (AnkG), which interacts with key ion channels, adhesion molecules, and cytoskeletal elements to stabilize AIS architecture. βIV Spectrin (βIVSpec), recruited by AnkG, reinforces the cytoskeletal framework, while voltage-gated sodium channels (Nav1.6) and potassium (K_v_1.2) channels localize to the AIS to regulate neuronal excitability (Huang and Rasband, 2018). Neurofascin 186 (NF186), a cell adhesion molecule, interacts with extracellular and intracellular components of the AIS and is critical for the initial clustering and maintenance of AnkG (Thaxton et al., 2010; Buttermore et al., 2012). While AnkG is indispensable for AIS formation and required for its long-term stability, NF186 has been shown to contribute to AIS maintenance and integrity, particularly in Purkinje cells. We and others have extensively characterized the individual roles of AnkG and NF186 in both nodal and AIS stabilization (Zhang et al., 1998; Koticha et al., 2006; Saifetiarova et al., 2017; Taylor et al., 2017). Conditional knockout studies have demonstrated the differential stability and functional roles of these proteins in the nodal and AIS domains. Specifically, loss of AnkG in adult neurons leads to progressive AIS deterioration and impaired neuronal signaling (Saifetiarova et al., 2017), whereas the absence of NF186 disrupts sodium channel clustering, destabilizing both nodes and the AIS (Taylor et al., 2017). Furthermore, simultaneous neuronal ablation of NF186 and AnkG in young and adult mice reveals an age-dependent increase in nodal stability in myelinated axons and differential effects on lifespan (Taylor et al., 2018). Despite substantial progress, the combined role of AnkG and NF186 in AIS assembly and their simultaneous loss during developmental stages in Purkinje cells remains unexplored.

While single-gene knockouts have revealed individual functions, the cooperative roles of NF186 and AnkG proteins in AIS maintenance, sodium channel clustering, and neuronal survival remain unexplored. Given that the AIS is a highly organized domain with multiple levels of molecular redundancy, it remains unclear whether NF186-dependent mechanisms can partially compensate for AnkG loss or whether the combined absence of both proteins leads to irreversible AIS destabilization. Importantly, because NF186 and AnkG occupy distinct positions within the AIS molecular hierarchy—NF186 being an extracellular adhesion protein and AnkG being an intracellular scaffolding protein—determining the impact of their single and combined loss at the AIS will provide a direct way to probe their individual and combined functions. To investigate the combined roles of AnkG and NF186 in Purkinje cell AIS organization and stability, we generated Purkinje cell-specific single- and double-knockout mouse models using *Pcp2-Cre*, which drives Cre recombinase expression predominantly in Purkinje cells during postnatal development. This approach allowed us to systematically analyze AIS disassembly over developmental stages, assess Na_v_1.6 channel clustering defects, evaluate the relationship between βIVSpec and AIS stability, and determine the impact on Purkinje neuron function. In parallel, we compared the double-knockout phenotype with every single knockout, enabling us to distinguish shared versus non-overlapping consequences of NF186 and AnkG loss at both the structural (AIS/pinceau) and transcriptomic levels. Our findings reveal that NF186 and AnkG play distinct yet complementary roles in maintaining AIS structure, ensuring sodium channel clustering, and preserving Purkinje cell excitability. Importantly, loss of AnkG had broader structural consequences for AIS stability than *NFKO*, in which AnkG remained present at the AIS and turned over slowly, whereas *AnkGKO* Purkinje cells showed progressive postnatal loss of NF186. These data indicate that *AnkGKO* phenocopies key aspects of the more severe double-knockout phenotype, as the combined loss of NF186 and AnkG resulted in more progressive AIS destabilization, culminating in Purkinje cell degeneration. Our results provide critical insights into the molecular mechanisms involving NF186 and AnkG in AIS assembly and maintenance, and axonal health, advancing our understanding of cerebellar dysfunction in neurodevelopmental and neurodegenerative diseases.

## Materials and methods

### Materials

All chemicals and reagents were purchased from Sigma Aldrich (St. Louis, MO), unless otherwise specified. Previously described antisera included rabbit anti-AnkG, anti-βIVSpec, and anti-pan Nav channels (Taylor et al., 2017), anti-Na_v_1.6 (#K87A,10, NeuroMab; Davis, CA) and anti-Calbindin (Sigma-Aldrich Cat# C9848), anti-Neurofilament (Covance Research Products Inc. Cat# SMI-312R), and anti-K_v_1.2 (#75–001, #75–380, #75–008, NeuroMab; Davis, CA). Fluorescent secondary antibodies used here were Alexa Fluor (Life Technologies, Grand Island, NY).

### Animals

*Nfasc^Flox^* (*NF^Fx^*) mice were reported previously (Pillai et al., 2009). *Ankyrin3^Flox^* (*AnkG^Fx^*) mice were generously provided by Vann Bennett (Duke University; Paez-Gonzalez et al., 2011). The *NF^Fx^* and *AnkG^Fx^* mice were bred to generate the *NF^Fx/Fx^*; *AnkG^Fx/Fx^* mouse line. This double floxed line was then crossed with *Pcp2-Cre* (Barski et al., 2000; Buttermore et al., 2012) to generate Purkinje cell-specific mutant lines, including *AnkGKO* (*Pcp2*; *AnkG^Fx/Fx^*), *NFKO* (*Pcp2*; *NFF^x/Fx^*), and *Pcp2*; *DKO* (*Pcp2-Cre*; *NF^Fx/Fx^*; *AnkG^Fx/Fx^*). Additionally, the double floxed line was crossed with *Parv-Cre* (Buttermore et al., 2012) to generate the Purkinje and basket cell-specific mutant mice: *Parv-Cre*; *NF^Fx/Fx^*; (*Parv*; *NFKO*), *Parv-Cre*; *AnkG^Fx/Fx^* (*Parv*; *AnkGKO*), and *Parv-Cre*; *NF^Fx/Fx^*; *AnkG^Fx/Fx^* (*Parv*; *DKO*) mice for analysis of basket cell/Purkinje cell-related pinceau phenotypes. Littermates or age-matched *NF^Fx/Fx^*; *AnkG^Fx/Fx^* mice lacking Cre were used as controls unless otherwise indicated.

All mice were maintained on a mixed strain *C57BL/6* and *129/SvEv* background. Mice were genotyped before postnatal day 10 (P10) and weaned by P21. All mice were group-housed in a temperature-controlled animal facility (23 ± 1 °C) with a maintained light cycle (12 h light on/12 h off) and *ad libitum* access to water and a standard rodent diet. An equal number of male and female mice were used in this study. Exact sample sizes, ages, and sex distribution for each experiment are provided in the corresponding figure legends. All animal research was performed with prior approval from the Institutional Animal Care and Use Committee of the University of Texas Health Science Center at San Antonio in accordance with the Public Health Service Policy on Humane Care and Use of Laboratory Animals.

### Motor balance and coordination assay

Motor coordination was measured using the edge-of-cage balancing test (Carter et al., 2001). We included at least five mice per genotype. Both male and female mice were used. Each mouse was gently placed onto the edge of the cage. The time each mouse stayed balanced without falling was recorded. A maximum duration of 60 s was allowed per trial. Each mouse completed three trials. Trials were performed once per day for three consecutive days. Motor function was measured using the Rotarod apparatus (Ugo Basile) as previously described (Steele et al., 2009). We used at least five mice per genotype. Both male and female mice were included in the study. Mice were trained on the Rotarod at 5 RPM for 5 min each day. Training was conducted for three consecutive days. During testing, the speed of the Rotarod increased gradually, as we described previously (Chang et al., 2023; Sell et al., 2025). Speed was increased from 5 RPM to 35 RPM over 2 min. We recorded the time until each mouse fell from the apparatus. Each mouse completed three trials.

### Immunofluorescence

Mice were anesthetized with an intraperitoneal injection of Avertin (2–2-2 tribromoethanol in 2-methyl-2-butanol). Once mice no longer responded to touch, mice were perfused intracardially using a peristaltic pump for 3 min with saline, followed by 2 min with a chilled 0.1 M phosphate buffer (PB) containing 4% PFA (2% PFA for Na_v_ staining). After perfusions, brains were carefully removed from each mouse and post-fixed overnight at 4 °C in 4% PFA solution (or 2% PFA for Na_v_ staining). After washing, tissues were placed in 30% sucrose in PBS at 4 °C until completely submerged (~2 days) and then cryopreserved at −80 °C. On the day of sectioning, the cerebella were embedded in optimal cutting temperature compound (Tissue-Tek) and cut along the sagittal plane at −20 °C into 20 μm sections using a Leica CM1860 cryostat. The sections were immediately placed on SuperFrost Plus glass slides (Fisher; Pittsburgh, PA) and immunostained as reported previously (Bhat et al., 2001; Shi et al., 2018; Sell et al., 2025). For immunostaining, tissues were processed from 3 to 4 mice per group per time point, then around 100 AIS from Purkinje cells from cerebella were quantified per animal at different ages. AIS regions were defined using Calbindin traces of the proximal axon from the base of the Purkinje soma.

### Image analysis

Confocal images of the cerebellum were acquired using a Zeiss LSM 710 Microscope using a 40x oil-immersion objective. Identical settings were maintained to capture images from control and mutant tissues. The representative immunofluorescence images shown are maximal intensity projections from Z-stacks with an interval of 0.4 μm. For quantification of immunofluorescence intensities, three z-stack images were taken for each mouse. Fluorescence intensity at the AIS was quantified using Fiji/ImageJ (NIH). The AIS was identified by (Ankyrin G or NF186 in single KO tissues, and Calbindin-positive Purkinje axons in the AIS area in the AnkG/NF double KO tissues. To account for local background noise and ensure technical robustness of individual data points, this information was used to calculate the corrected fluorescence for each AIS: integrated density - (area of selected AIS x mean fluorescence of background readings). For each image, three independent background readings were taken from non-specific regions within the same field of view to obtain a precise mean background value. This high-resolution, background-corrected approach allowed for the assessment of protein distribution across thousands of individual axonal segments while maintaining biological stringency across independent replicates.

To evaluate the spatial distribution of the K_v_1.2 signal along Purkinje cell axons, we performed fluorescence intensity line profile analysis using ImageJ. Confocal images were captured from parasagittal cerebellar sections of control *NF^Fx/Fx^*; *AnkG^Fx/Fx^* and mutant mice at postnatal day 10, 20, and 30 (P10, P20, and P30). Calbindin immunostaining was used to visualize Purkinje cell bodies and axons, while K_v_1.2 labeling defined the pinceau structure. A straight line (~30–40 μm) was drawn from the base of the soma along the AIS toward the pinceau. Using the “Plot Profile” function in ImageJ, we extracted pixel intensity values for K_v_1.2 along the line. Data from multiple cells (n = 3 AISs per animal, 3 animals per group) were exported and averaged for each genotype. Intensity values were normalized to the peak signal within each line for comparison of distribution shape and localization.

### RNA sequencing and bioinformatic analysis

Cerebellar hemispheres were dissected from P32 litter-mates from *NF^Fx/Fx^*; *AnkG^Fx/Fx^* (control), *Pcp2-Cre*; *NFF^x/Fx^*, (*NFKO*; *n* = 3), *Pcp2-Cre*; *AnkG^Fx/Fx^* (*AnkGKO*; *n* = 3), and *Pcp2-Cre*; *NFF^x/Fx^*; *AnkG^Fx/Fx^* (*Pcp2*; *DKO*; *n* = 3), and the single or double floxed alleles without Cre (Controls; *n* = 3). Tissues were flash-frozen in liquid N₂, and total RNA was extracted with the RNeasy Plus Mini kit (Qiagen) following the manufacturer’s protocol, including on-column DNase digestion. RNA integrity was assessed on an Agilent 4,200 TapeStation; all samples exhibited an RNA integrity number (RIN) ≥ 8.2. Poly-adenylated RNA was enriched (NEBNext Poly(A) mRNA Magnetic Isolation Module), and strand-specific libraries were prepared with the NEBNext Ultra II Directional RNA Library Prep kit using unique dual indices. Libraries were pooled equimolarly and sequenced on an Illumina NovaSeq 6,000 S4 flow cell (paired-end 150 bp). Each sample yielded 38–44 million read-pairs (mean ± SD: 41.2 ± 2.1 M); ≥ 93% of bases exceeded Q 30.

Raw reads were inspected with FastQC v0.12.1, trimmed with Trimmomatic v0.39 (ILLUMINACLIP:2:30:10 SLIDINGWINDOW:4:20 MINLEN:36), and aligned to the *Mus musculus* reference genome GRCm39 (Ensembl release 111) with STAR v2.7.11a (—twopassMode Basic,—outSAMtype BAM SortedByCoordinate). Alignment rates were 91–95%. Gene-level counts were generated during mapping with STAR’s—quantMode. GeneCounts option and imported into R 4.3.2. Downstream analyses used Bioconductor 3.18 packages. Genes with < 10 total counts across all libraries were excluded, leaving 17,621 features.

Differential expression was calculated with DESeq2 v1.40.2 (design = ~ condition). Size factors were estimated by the median-ratio method; dispersion was fit with the default local model. Wald statistics were subjected to Benjamini–Hochberg correction; transcripts with adjusted *p* < 0.05 and |log₂(fold-change)| ≥ 0.6 were considered significant. Log₂-fold-changes plotted in the manuscript were shrinkage-estimated with apeglm v1.22.0. Variance-stabilized data (vst) were used for principal-component analysis (DESeq2:plotPCA) and heatmaps (pheatmap v1.0.12; color scale RdBu, 255 breaks). Gene-ontology enrichment for Biological Process was performed with clusterProfiler v4.10.1 (OrgDb = org. Mm.eg.db v3.18.0; pAdjustMethod = “BH”; q-value < 0.05). Dot plots were generated with enrichplot v1.22.0. Kyoto Encyclopedia of Genes and Genomes (KEGG) pathways were queried with clusterProfiler:enrichKEGG (organism = “mmu”), and g: Profiler2 v0.2.1 was used for the secondary enrichment of STRING networks. Protein–protein interaction analysis employed STRINGdb v2.6.5 (species = 10,090; version = 12.0; scoreThreshold = 700). Networks were exported to Cytoscape 3.10.1 for figure preparation. All statistical tests are two-tailed; specific thresholds are reported in figure legends. Sequencing data have been deposited in GEO (accession GSE322737).

### Statistical analysis

**Figure 1:**
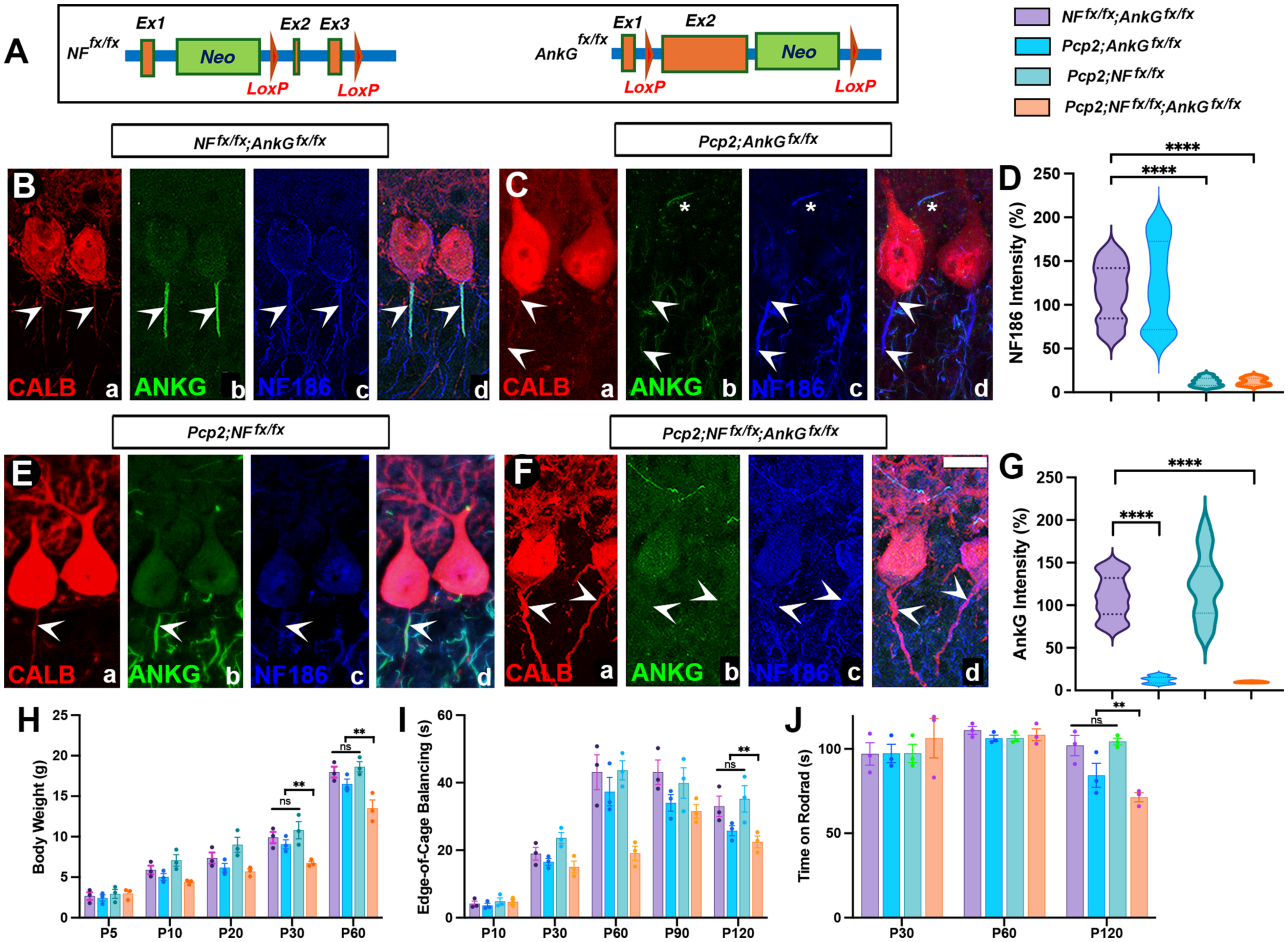
Purkinje cell-specific ablation of Neurofascin and Ankyrin G. **(A)** Schematics depicting the location of *loxP* sites in *Nfasc* (*NF^Fx/Fx^*) and *AnkG* (*AnkG^Fx/Fx^*). **(B,C,E,F)** Control *NF^Fx/Fx^*; A*nkG^Fx/Fx^* **(B)** and Purkinje-specific ablation of *AnkG* in *Pcp2-Cre*; *AnkG^Fx/Fx^* **(C)**, *Nfasc* in *Pcp2-Cre*; *NFF^x/Fx^* **(E)**, and AnkG and NF186 in *Pcp2-Cre*; *NFF^x/Fx^*; *AnkG^Fx/Fx^* **(F)**. Cerebellar sections from P15 mice immunostained against NF186 (blue), AnkG (green), and calbindin (red). Note that Nfasc and AnkG are localized to the Purkinje AIS (Bb, Bc, arrowheads) in the control sections. AnkG is absent in *Pcp2-Cre*; *AnkG^Fx/F^* (Cb), and NF186 is absent in *Pcp2-Cre*; *NFF^x/Fx^* (Ec), and both are absent in the *Pcp2-Cre*; *NFF^x/Fx^*; *AnkG^Fx/Fx^* sections (Fb, Fc, arrowheads). Note that at P15 NF186 is present at the AIS from *Pcp2-Cre*; *AnkG^Fx/Fx^*(Cc), and AnkG is present at the AIS in *Pcp2-Cre*; *NFF^x/Fx^* (Eb). Note the basket cell AIS is also visible (Cb–d, asterisks). Scale bar = 10 μm. **(D,G)** Immunofluorescence intensity quantification of NF186 **(D)** and AnkG **(G)** at the AIS from all genotypes, representing 150–200 individual AISs per animal from 3 independent animals. Statistical significance was determined based on the mean value of each animal. **(H)** Body weight measurements of mice from P5 to P60 in all genotypes (*n* = 3). **(I)** Edge of cage balan cing by all genotypes from P10 to P120 (*n* = 3). **(J)** Time on the rotarod for all genotypes at P30, P60, and P120 (*n* = 3). All data are represented as mean ± SEM, *p < 0.05, **p < 0.01, ***p <  0.001 by one-way ANOVA (comparing mutants with control).

**Figure 2:**
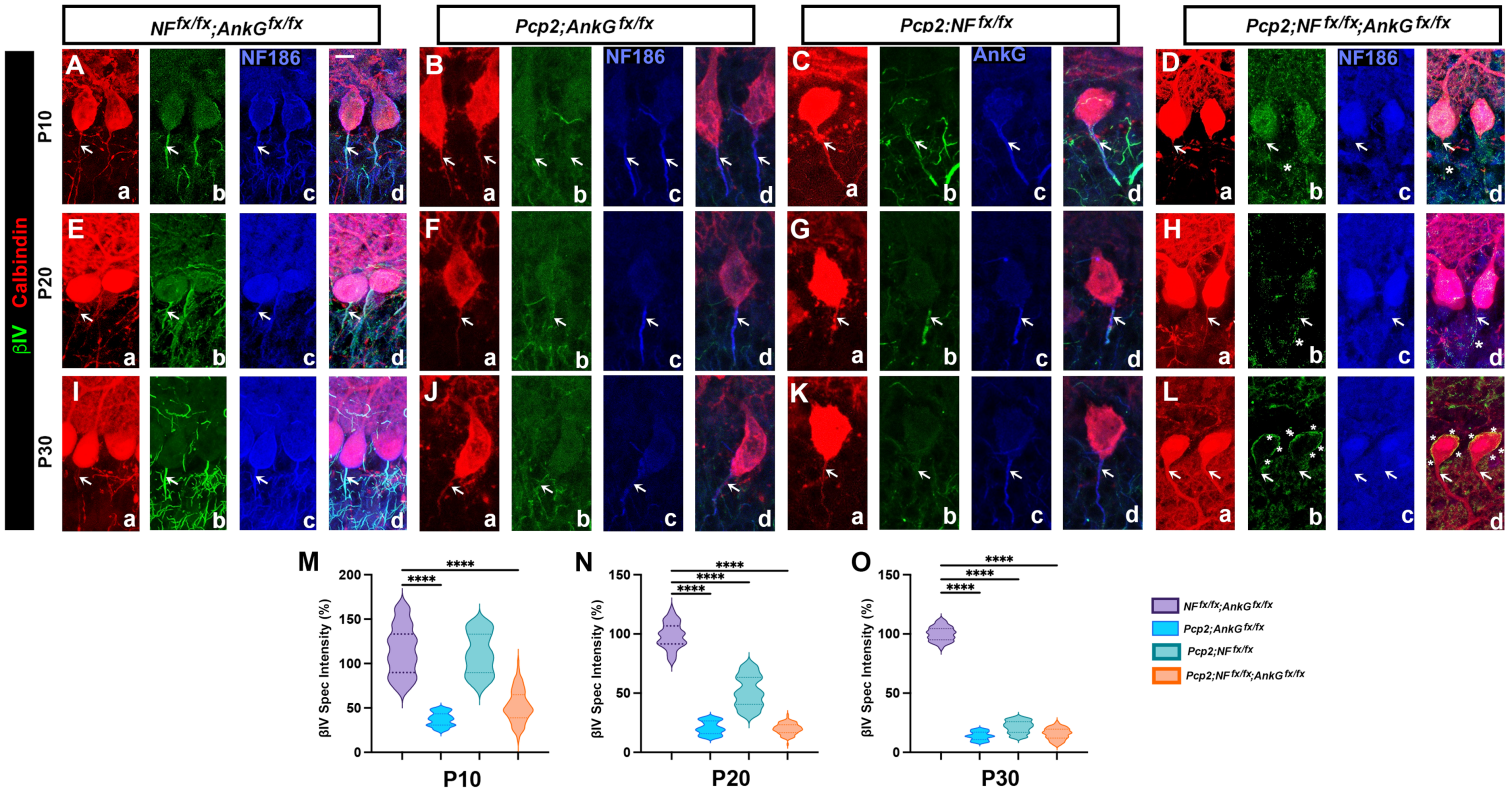
*β*IV Spectrin clustering at the axon initial segment in Neurofascin and Ankyrin G single and double mutants. **(A–L)** Cerebellar sections from control *NF^Fx/Fx^*; A*nkG^Fx/Fx^*, *Pcp2-Cre*; *AnkG^Fx/Fx^*, *Pcp2-Cre*; *NFF*^*x/Fx*,^ and *Pcp2-Cre*; *NFF^x/Fx^*; *AnkG^Fx/Fx^* mice at P10 **(A–D)**, P20 **(E–H)**, and P30 **(I–L)** immunostained against AnkG (blue in **C,G,K**) or NF186 (blue in **A,B,D,E,F,H,I,J,L**), βIV Spectrin (βIV; green) and calbindin (red). Note that βIV is properly localized at the Purkinje AIS (Ab, Eb, Ib, arrow) in the control sections at all postnatal time points but is either degraded or absent in the other mutant sections. Asterisks in Db, d and Hb, d point to remaining βIV staining. Asterisks in Lb, d point to localization of βIV in Purkinje cell membrane. Scale bar, 10 μm. **(M–O)** Immunofluorescence intensity quantification of βIV at the AIS in all four genotypes at P10 (**M)**, P20 **(N)**, and P30 **(O)**, representing 150–200 individual AISs per animal from 3 independent animals. Statistical significance was determined based on the mean value of each animal. All data are represented as mean ± SEM. *p < 0.05, **p < 0.01, ***p < 0.001 by one-way ANOVA analysis (comparing mutants with control).

**Figure 3:**
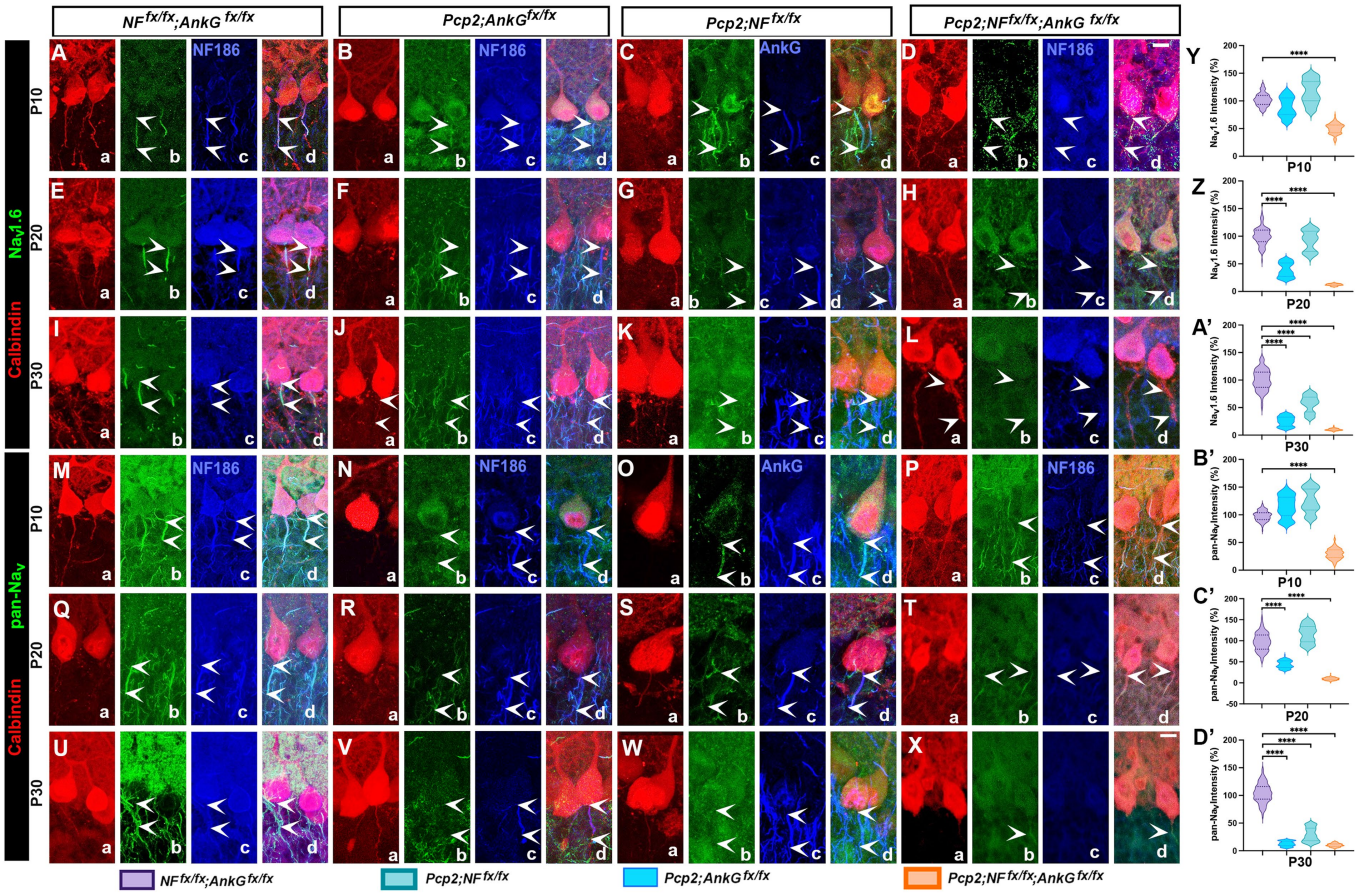
Proper localization and maturation of sodium channels is severely affected at the axon initial segment in the absence of Neurofascin and Ankyrin G. **(A–L)** Immunofluorescence images of Na_v_1.6 channel expression in the AIS of control *NF^Fx/Fx^*; A*nkG^Fx/Fx^* **(A,E,I)**, *Pcp2-Cre*; *AnkG^Fx/Fx^* **(B,F,J)**, *Pcp2-Cre*; *NFF^x/Fx^* **(C,G,K)**, and *Pcp2-Cre*; *NFF^x/Fx^*; *AnkG^Fx/Fx^* **(D,H,L)** mice at P10 **(A–D)**, P20 **(E–H)**, and P30 **(I–L)**. Arrowheads indicate the location of AIS in control and other mutants. Scale bars: 10 μm. **(M–X)** Immunofluorescence images of pan-Na_v_ channel expression at the AIS of control *NF^Fx/Fx^*; A*nkG^Fx/Fx^* **(M,Q,U)**, *Pcp2-Cre*; *AnkG^Fx/Fx^* **(N,R,V)**, *Pcp2-Cre*; *NFF^x/Fx^* **(O,S,W)**, and *Pcp2-Cre*; *NFF^x/Fx^*; *AnkG^Fx/Fx^* **(P,T,X)** mice at P10 **(M–P)**, P20 **(Q–T)**, and P30 **(U–X)**. Arrowheads indicate the location of AIS in control and other mutants. Scale bars: 10 μm. **(Y,Z,A’)** Immunofluorescence intensity quantification of Na_v_1.6 channels at the AIS in all four genotypes at P10 **(Y)**, P20 **(Z)** and P30 **(A’)**, representing 150–200 individual AISs per animal from 3 independent animals). Statistical significance was determined based on the mean value of each animal. **(B’–D′)** Immunofluorescence intensity quantification of pan-Na_v_ channels at the AIS in all four genotypes at P10 **(B′)**, P20 **(C′)** and P30 **(D′)**, representing 150–200 individual AISs per animal from 3 independent animals). Statistical significance was determined based on the mean value of each animal. All data are represented as mean ± SEM. ****p < 0.0001 by one-way ANOVA analysis (mutants comparing mutants with controls).

**Figure 4:**
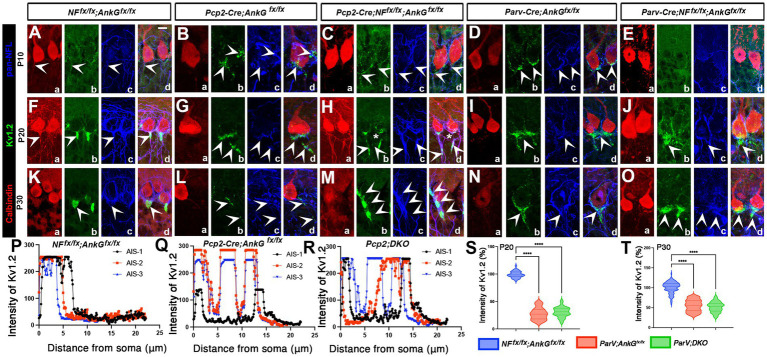
Pinceau organization at the Purkinje cells AIS is disrupted in the absence of Neurofascin and Ankyrin G. **(A–O)** Control *NF^Fx/Fx^*; A*nkG^Fx/Fx^* **(A,F,K)**, *Pcp2-Cre*; *AnkG^Fx/Fx^* **(B,G,L)**, *Pcp2-Cre*; *NFF^x/Fx^*; *AnkG^Fx/Fx^* **(C,H,M)**, *Parv-Cre*; *AnkG^Fx/Fx^* **(D,I,N)**, and *Parv-Cre*; *NFF^x/Fx^*; *AnkG^Fx/Fx^* **(E,J,O)** cerebellar sections from P10 **(A–E)**, P20 **(F–J)**, and P30 **(K–O)** mice immunostained against pan-Neurofilament, pan-NFL (blue), K_v_1.2 (green), and calbindin (red). Arrowheads indicate the location of pinceau in controls or K_v_1.2 staining and clusters at the AIS/pinceau region. Scale bars: 10 μm. **(P–R)** Line intensity profiles of K_v_1.2 fluorescence along the AIS in control *NF^Fx/Fx^*; A*nkG^Fx/Fx^* **(P)**, *Pcp2-Cre*; *AnkG^Fx/Fx^* **(Q)**, and *Pcp2-Cre*; *NFF^x/Fx^*; *AnkG^Fx/Fx^* (*Pcp2*; *DKO*), **(R)** Purkinje cells at P20. Profiles were generated using ImageJ plot profile analysis along axonal segments extending from the soma to the end of the pinceau. **(S,T)** Quantification of total K_v_1.2 fluorescence intensity in the pinceau region at P20 (S) and P30 (T). Values were obtained from maximum intensity projections of immunostained sections and normalized to control levels, representing 150–200 individual AISs per animal from 3 independent animals. Statistical significance was determined based on the mean value of each animal. All data are shown as mean ± SEM; ****p < < 0.0001 by one-way ANOVA analysis comparing mutants with control.

All data are presented as mean ± SEM unless otherwise indicated. For animal-based experiments, n refers to the number of mice per genotype. For imaging-based quantifications, n is defined in the corresponding figure legends and represents the number of analyzed AISs, cells, or animals, as appropriate. Specifically, in the violin plots presented in Figures 1–4, individual data points (150–200 AIS per animal) represent expression levels from single AIS staining to visualize cellular heterogeneity. However, to ensure biological robustness and avoid the inflation of *p*-values through pseudoreplication, statistical significance was determined using the mean values derived from *n* = 3 biological replicates (mice) per experimental group. All n-numbers, representing both total nuclei quantified and independent biological replicates, are explicitly labeled in the corresponding figure legends. Statistical analyses were performed using GraphPad Prism 10 software (GraphPad Software, San Diego, CA). For comparisons involving multiple genotypes and/or ages, one-way ANOVAs were performed, followed by Tukey’s comparison analyses. When only a single time point was used, statistically significant differences between two genotypes were determined using unpaired, two-tailed Student’s *t*-tests. Statistical differences are represented in figures by * (*p* < 0.05), ** (*p* < 0.01), *** (*p* < 0.001), **** (*p* < 0.0001) with black asterisks indicating differences between age-matched control and mutants. Exact statistical tests, sample sizes, and definitions of n for each experiment are provided in the corresponding figure legends.

## Results

### Ablation of Neurofascin and Ankyrin G in Purkinje cells results in progressive motor impairment and ataxia

Previous studies have established the role of Neurofascin 186 (NF186) in Purkinje and basket neurons to coordinate cerebellar pinceau organization during postnatal development (Buttermore et al., 2012). In addition to NF186, the cytoskeletal scaffolding protein Ankyrin G (AnkG) is also expressed at the nodes of Ranvier and at the AIS (Thaxton et al., 2011; Jenkins et al., 2015; He et al., 2022). We wanted to investigate the combined roles of NF186 and AnkG in Purkinje cells on the developmental organization of the Purkinje cell AIS and the pinceau that forms at the AIS. To accomplish this, we generated Purkinje cell-specific Neurofascin (NF186) and Ankyrin G (AnkG) single (*NFKO* and *AnkGKO*) and double (*Pcp2*; *DKO*) knockout mice. Figure 1A illustrates the *Nfasc* (Pillai et al., 2009) and *AnkG* (Paez-Gonzalez et al., 2011) mouse lines we employed to create the mutant mice. To confirm the effective deletion of both NF186 and AnkG in Purkinje cells, we performed immunofluorescence analyses of control (*NF^Fx/Fx^*; *AnkG^Fx/Fx^*), every single knockout (*Pcp2-Cre*; *AnkG^Fx/Fx^* or *Pcp2-Cre*; *NFF^x/Fx^*), and *Pcp2*; *DKO* at postnatal day 15 (P15) using anti-NF186, anti-AnkG antibodies to label the AIS and anti-Calbindin to label the Purkinje cells (Figures 1B,C,E,F). As shown in Figure 1B, Purkinje cells from control *NF^Fx/Fx^*; *AnkG^Fx/Fx^* mice exhibited robust expression of both NF186 and AnkG at the AIS (arrowheads). Purkinje cells from *AnkGKO* lacked AnkG (Figure 1Cb, arrowheads) but retained NF186 at the AIS, and *NFKO* mice lacked NF186 (Figure 1Ec, arrowheads) and retained AnkG. The *Pcp2*; *DKO* mice displayed a complete absence of NF186 and AnkG immunoreactivity at P15, confirming successful ablation of NF186 and AnkG (Figure 1F, arrowheads). Quantification of NF186 (Figure 1D) and AnkG (Figure 1G) fluorescence intensity in AnkG and NF186 single and double mutants using violin plots showed significant loss of AnkG and NF186 in corresponding single knockouts (Figures 1C,E) as well as loss of both AnkG and NF186 in double mutants (Figure 1F) compared to controls. These data confirm efficient ablation of AnkG and NF186 in Purkinje cells and indicate a slow turnover of proteins at the AIS.

We next asked whether disrupting NF186 and/or AnkG in Purkinje cells affects growth and motor behavior. Longitudinal body weight measurements from P5 to P60 revealed no significant differences between controls and either of the single knockouts at any time point, including P60 (Figure 1H). In contrast, *Pcp2*; *DKO* mice began to diverge after P30 and showed a significant reduction in body weight by P60 (Figure 1H) compared to controls, indicating that combined NF186 and AnkG loss affects postnatal growth. Consistent with this pattern, motor coordination assessed by the edge-of-cage balancing assay showed progressive improvement in control mice across development, while *Pcp2*; *DKO* mice exhibited a clear deficit emerging at P30 that persisted into adulthood (Figure 1I). Notably, single *NFKO* and *AnkGKO* mice did not show statistically significant impairments compared to controls. However, *AnkGKO* mice displayed a trend toward reduced performance at later time points (Figure 1I), suggesting that AnkG loss may have a stronger functional impact than NF186 loss when each is removed individually. Similarly, in the accelerating rotarod assay, *Pcp2*; *DKO* mice showed impaired performance at later ages (Figure 1J), whereas single knockouts did not differ significantly from controls, with a modest downward trend in *AnkGKO* mice (Figure 1J). Taken together, the absence of significant body weight loss and behavioral deficits in either single knockout—contrasted with the robust impairments in the double knockout—supports a synthetic interaction between NF186 and AnkG in maintaining cerebellar-dependent motor function.

### βIV Spectrin fails to cluster at the Purkinje cell axon initial segment in the absence of Neurofascin and/or Ankyrin G

βIV Spectrin (βIVSpec) is an essential cytoskeletal scaffolding protein that is highly enriched at the neuronal AIS and the nodes of Ranvier in myelinated axons (Koticha et al., 2006; Thaxton et al., 2011). βIVSpec forms a periodic lattice with the actin cytoskeleton, which underlies the axonal membrane scaffold (Komada, 2002; Ho et al., 2014). In healthy neurons, βIVSpec is recruited to the AIS by AnkG and stabilizes resident ion channels and other proteins. To determine how loss of NF186 and/or AnkG impacts the AIS cytoskeleton during development, we examined βIVSpec localization in Purkinje neurons from control, *AnkGKO* (*Pcp2*; *AnkG^Fx/Fx^*), *NFKO* (*Pcp2*; *NF^Fx/Fx^*), and double knockout (Pcp2; DKO) mice at P10 (early AIS formation), P20 (AIS maturation), and P30 (established AIS; Figures 2A–L). Cerebellar sections were immunostained for βIVSpec (green), together with calbindin (red), to label Purkinje cells; NF186 or AnkG staining (blue) was included as indicated to visualize the targeted AIS component in each genotype (Figures 2A–L). In control Purkinje cells, strong βIVSpec immunoreactivity marked a continuous AIS segment at all ages examined (Figures 2A,E,I; arrows). At P10, βIVSpec was already detectable as a defined stretch distal to the soma, and by P20 and P30 it was intensely concentrated within a well-defined AIS, consistent with normal maturation of the AIS cytoskeletal scaffold (Figures 2A,E,I). In *AnkGKO* mice, βIVSpec clustering showed severe impairment. At P10, AIS-associated βIVSpec signal was very weak and poorly defined in *AnkGKO* Purkinje cells (Figure 2Bb; arrows, quantified in 2 M), and this deficiency persisted at P20 and P30, with βIVSpec remaining faint and lacking a clear AIS-enriched segment (Figures 2Fb,Jb; arrows, quantified in 2 N, 2O). In *NFKO* mice, βIVSpec initially appeared largely preserved at P10, with a clear AIS-associated βIVSpec signal comparable in overall pattern to controls (Figure 2Cc; arrows, quantified in 2 M). However, by P20, the βIVSpec signal at the AIS was noticeably diminished and less sharply confined (Figure 2Gc; arrows, quantified in 2 N), and by P30 it was nearly absent from the proximal axon, approaching background levels (Figure 2Kc; arrows, quantified in 2O). This temporal progression indicates that NF186 loss permits early βIVSpec accumulation but fails to sustain βIVSpec enrichment at the AIS during maturation, culminating in near-complete loss of AIS βIVSpec by P30. In the double knockout, βIVSpec signal was minimal within the proximal axon and often appeared diffuse or redistributed relative to the control AIS pattern, consistent with profound destabilization of the AIS cytoskeletal scaffold when both NF186 and AnkG are removed (Figures 2D,H,L). At P10, some *Pcp2*; *DKO* Purkinje cells exhibited punctate staining of βIVSpec in the proximal axon. Unlike the cohesive AIS seen in the control Purkinje cells, this staining was often discontinuous or fragmented. Rather than a single cohesive segment, at P10 βIVSpec appeared as punctate clusters in the proximal axon (Figure 2Db, arrows, quantified in 2 M). Quantitative analysis confirmed a significantly lower AIS/soma βIVSpec fluorescence ratio in *Pcp2*; *DKO* vs. control at P10 (*p* < 0.01), indicating that initial recruitment of Spectrin to the AIS was already impaired. By P20, the difference became more pronounced in *Pcp2*; *DKO* Purkinje cells as they failed to maintain any discrete βIVSpec-positive AIS. Most Purkinje axons in the *Pcp-2*; *DKO* showed only diffuse βIVSpec labeling along the axon, with no clear start-stop boundary near the soma (Figure 2Hb, arrows, quantified in 2 N, P20). The small puncta of βIVSpec seen at P10 were largely lost by P20, suggesting a progressive disassembly of the axonal spectrin scaffold at the AIS in the absence of NF186 and AnkG. At P30, βIVSpec was virtually undetectable in the proximal axons of *Pcp2*; *DKO* Purkinje cells and was indistinguishable from that of the background (Figure 2Lb, quantified in 2O, P30). βIVSpec immunoreactivity was observed in the soma cell membrane and not at the AIS, indicating a redistribution of βIVSpec in the absence of both NF186 and AnkG (Figures 2Lb,d, asterisks), which was not observed in AnkG and NF186 single mutants even at P30 (Figures 2Jb,Kb). Together, these data indicate that AnkG is required for robust βIVSpec clustering at the Purkinje AIS from the earliest postnatal stages, whereas NF186 is more critical for maintaining/stabilizing βIVSpec enrichment during AIS maturation, with progressive loss becoming most apparent between P20 and P30. However, the combined absence of both NF186 and AnkG leads to defects in both the organization and stabilization of the AIS and misdistribution of βIVSpec to Purkinje cell soma.

### Loss of NF186 and AnkG causes failure of sodium channels to cluster at the Purkinje cell axon initial segment

High-density clustering of voltage-gated sodium (Na_v_) channels at the AIS is crucial for lowering the threshold and initiation of action potentials in Purkinje neurons (Wang et al., 2017; Leterrier et al., 2010). In mature Purkinje cells, Na_v_1.6 is the predominant sodium channel isoform at the AIS, replacing Na_v_1.2 after the second postnatal week as the cerebellum matures (Buttermore et al., 2012; Freal and Hoogenraad, 2025). We therefore examined whether the disruption of the AIS scaffold in NF186/AnkG single or double mutants affected the localization of Na_v_ channels in Purkinje cells over time using two markers: (i) an Na_v_1.6-specific antibody to specifically assess whether Na_v_1.6 channel clusters at the Purkinje cell AIS (Figures 3A–L) and (ii) a pan-Na_v_ antibody that recognizes all sodium channel isoforms concentrated at the AIS (Figures 3M–X).

Immunostaining specifically against Na_v_1.6 in control mice showed robust sodium channel clustering that was largely restricted to the AIS, consistent with proper targeting of the Na_v_1.6 channel to the AIS during maturation (Figures 3Ab,Eb,Ib, arrowheads). Co-labeling for AnkG in control cells confirmed that Na_v_ 1.6 channels colocalized with the AnkG-defined AIS segment, and line-scan analysis showed a steep rise in Na_v_ signal at the AIS start, with the signal plateauing along its length. At P10, *AnkGKO* mutants showed a relatively normal distribution of Na_v_1.6 that corresponded with the localization of NF186 (Figure 3Bb, arrowheads). At P20 and P30, Na_v_1.6 was undetectable at the AIS (Figures 3Fb,Jb, arrowheads), indicating that Na_v_1.6 fails to remain at the AIS in the absence of AnkG. Interestingly, NF186 was normally localized to the AIS at P10 and P20 in *AnkGKO* mutants but was undetectable at P30, indicating that NF186 maintenance of NF186 at the AIS requires AnkG (Figures 3Bc,Fc,Jc, arrowheads). In *NFKO* mutants, Na_v_1.6 showed normal distribution that corresponded with the localization of AnkG at P10 (Figure 3Cb, arrowheads). At P20 and P30, Na_v_1.6 was still detectable, but the levels were severely reduced at the AIS (Figures 3Gb,Kb, arrowheads), indicating that Na_v_1.6 fails to remain properly at the AIS in the absence of NF186. Interestingly, AnkG was detectable at the AIS in *NFKO* mice at P10 but failed to fully maintain Na_v_1.6 at the AIS (Figures 3Gc,Kc, arrowheads). In *Pcp2*; *DKO* Purkinje cells, Na_v_1.6 failed to properly localize to the AIS (Figures 3Db,Hb,Lb, arrowheads indicate the starting point of the axon). In control Purkinje cells at P10, Na_v_1.6 was already getting enriched in the AIS (Figure 3Ab, arrowheads), whereas in DKO cells at P10, Na_v_1.6 staining was weak and often seen in the soma or dispersed in the proximal axon without forming a cluster (Figures 3Db,d, arrowheads). In control Purkinje cells at P20 (Figure 3Eb, arrowheads) and P30 (Figure 3Ib, arrowheads), Na_v_1.6 was highly enriched in the AIS; whereas, in DKO cells, Na_v_1.6 staining was undetectable at the AIS at both P20 (Figures 3Hb,d, arrowheads) and P30 (Figures 3Lb,d, arrowheads). These data show that NF186 loss permits early Na_v_1.6 accumulation but fails to maintain mature Na_v_1.6 clustering during AIS maturation, and that loss of AnkG severely reduced Na_v_1.6 clustering at the AIS and its maintenance even in the detectable presence of NF186. However, loss of both AnkG and NF186 more severely affected the initial organization and the maintenance of Na_v_1.6 at the AIS, which was evident at P10 and later time points.

Next, we investigated whether other sodium channels (e.g., Na_v_1.2) might be present at the AIS in the mutants in the absence of Na_v_1.6 channels using pan-Nav immunostaining. In control mice, at P10, pan-Na_v_ immunostaining already revealed a focused accumulation of Na_v_ channels at the AIS of most Purkinje cells (Figure 3Mb), reflecting early Na_v_1.2 enrichment. By P20 and P30, the AIS staining intensity for pan-Na_v_ increased further, and the immunoreactivity became prominent at the AIS (Figures 3Qb,Ub). Consistent with the Na_v_1.6 results, the single knockout comparisons showed that *AnkGKO* Purkinje cells exhibited very weak AIS-associated pan-Na_v_ staining beginning at P10 (Figure 3Nb, arrowheads), which was further compromised at P20 and P30 (Figures 3Rb,Vb, arrowheads); however, NF186 was still detectable at the AIS (Figures 3Nc,Rc,Vc, arrowheads). *NFKO* Purkinje cells showed AIS-associated pan-Na_v_ signal at P10 (Figure 3Ob, arrowheads), which was severely reduced by P20 (Figure 3Sb, arrowheads) and by P30. Na_v_ immunoreactivity was essentially undetectable at the AIS (Figure 3Wb, arrowheads). In *NFKO* AISs, AnkG was still detectable at the AIS (Figures 3Oc,Sc,Wc, arrowheads). The NF186/AnkG double mutant Purkinje cells exhibited a dramatic loss of sodium channel clustering at the AIS. At P10, when control AIS showed clear Na_v_ immunoreactivity, *Pcp2*; *DKO* Purkinje cells showed diminished and diffuse pan-Na_v_ staining. Some DKO cells had a faint AIS region labeling (Figure 3Pb, asterisk), but many showed Na_v_ channels distributed more evenly throughout the soma and proximal axon. By P20 (Figure 3Tb, asterisk) and P30 (Figure 3Xb, arrowhead), the difference was stark: whereas control Purkinje AIS showed robust clustering of Na_v_ channels, DKO Purkinje cells had no discernible Na_v_ clusters at the AIS that closely resembled the DKO pattern of the Na_v_1.6 mislocalization at the AIS.

Quantification of the AIS/soma immunofluorescence ratio for Na_v_1.6 and pan-Na_v_ channels in control and mutants at each developmental stage analyzed is shown in Figures 3Y,B’ for P10, Figures 3Z,C’’ for P20, and Figures 3A’,D’ for P30. Na_v_1.6 fluorescence intensity at the AIS was significantly reduced in DKO mice compared with controls across developmental stages analyzed, with *AnkGKO* exhibiting strong reductions from P10 onward, whereas *NFKO* showed a delayed decline that became pronounced at P20 and P30. Similarly, quantification of pan-Na_v_ AIS enrichment demonstrated strong reductions in *AnkGKO* and *DKO* at all time points, with *NFKO* showing a progressive loss that approached DKO levels by P30. Together, these results indicate that AnkG is required for robust early clustering of sodium channels at the Purkinje AIS. At the same time, NF186 contributes prominently to the stabilization and maintenance of Na_v_ channel enrichment during AIS maturation, such that combined loss produces the most severe and persistent failure of Na_v_ channel accumulation at the AIS.

### Basket cell pinceau formation is severely impaired in the absence of Neurofascin 186 and Ankyrin G

The pinceau is a specialized axo-axonic structure formed by basket cell terminals enveloping the AIS of Purkinje cells and is essential for inhibitory regulation and cerebellar output (Buttermore et al., 2012). To better understand how the AIS and presynaptic basket cell projections coordinate to establish this structure, we examined the localization of K_v_1.2, a key potassium channel enriched in the pinceau, in cell-type-specific NF186/AnkG knockout models across developmental stages. We carried out immunofluorescence staining with antibodies against calbindin (Figure 4, red), K_v_1.2 (green), and Neurofilaments (pan-NFL, Blue). We first analyzed control cerebellar sections from P10 to P30 (Figures 4A,F,K). At P10, K_v_1.2 staining was weak or absent near the Purkinje AIS, and mostly observed in basket cell terminals that target the Purkinje cell soma and the AIS (Figure 4Ab, arrowheads), consistent with the known timeline of pinceau maturation (Buttermore et al., 2012). By P20, robust K_v_1.2 immunoreactivity was observed in compact clusters at the base of Calbindin-positive Purkinje soma around the AIS (Figure 4Fb, arrowhead). The pan-NFL staining highlighted the basket cell terminals and Purkinje cell axons. At P30, the pinceau was well defined, with tightly organized K_v_1.2-positive basket cell terminals enveloping the proximal axon at the AIS (Figure 4Kb, arrowhead). In Purkinje cell-specific *AnkGKO* mice, K_v_1.2 mislocalization defects were evident but generally less severe than in the Pcp2; DKO mice. At P10, K_v_1.2 localization around the AIS region appeared largely comparable to controls (Figure 4Bb compared with Figure 4Ab), indicating that early K_v_1.2 recruitment to developing basket terminals can occur despite loss of AnkG in Purkinje cells. By P20, however, K_v_1.2 clustering was reduced and less sharply confined to the distal AIS region (Figure 4Gb, arrowheads), with greater signal dispersion compared with controls (compare Figure 4Fb with Figure 4Gb). At P30, K_v_1.2 remained disorganized and failed to form a compact pinceau structure in many Purkinje cells (Figure 4Lb compare with Figure 4Kb, arrowheads). Consistent with these observations, line profile analysis showed that *AnkGKO* mice exhibited a reduced distal AIS peak and broader K_v_1.2 distribution relative to controls (Figure 4Q, compare with Figure 4P). Together, these data indicate that loss of AnkG in Purkinje cells is sufficient to impair maturation and spatial confinement of K_v_1.2-rich basket terminals. In Purkinje cell-specific double knockout mice, *Pcp2-Cre*; *NF^Fx/Fx^*; *AnkG^Fx/Fx^* (*Pcp2*; *DKO*), K_v_1.2 localization was notably altered. At P10, K_v_1.2 expression and localization did not appear significantly different from those of control tissues (compare Figure 4Ab with Figure 4Cb, arrowheads). At P20 (Figure 4Hb, arrowheads), K_v_1.2 staining was present but appeared fragmented and often mislocalized to distal portions of the AIS (Figure 4Hb, arrowheads, compare with Figure 4Fb control). By P30, clustering remained incomplete, with more diffuse and disorganized K_v_1.2 clusters that failed to form a typical pinceau structure around the Purkinje cell AIS (Figure 4Mb, compare with Figure 4Kb, arrowheads). We compared the K_v_1.2 fluorescence distribution in Purkinje axons between control and *Pcp2*; *DKO* mice using line profile analysis (Figure 4R, showing a representative AIS analysis for P20). In control mice (Figure 4P), K_v_1.2 intensity exhibited a distinct peak at the distal AIS, corresponding to the pinceau structure, with little signal observed in the proximal axon. In contrast, *Pcp2*; *DKO* mice (Figure 4R) showed a broader Kv1.2 signal spread along the AIS, with reduced peak intensity at the pinceau and increased proximal labeling. Together, these data indicate that loss of AnkG in Purkinje cells is sufficient to impair maturation and spatial confinement of K_v_1.2-rich basket terminals, and that combined loss of NF186 and AnkG produces a stronger disruption of pinceau organization.

We have previously shown that loss of NF186 in Purkinje and basket cells affects the organization of pinceau at the Purkinje AIS (Buttermore et al., 2012). To determine the contribution of Purkinje and basket cell AnkG in pinceau organization, we generated Parvalbumin-Cre-driven *AnkG* knockouts. As shown in Figure 4Db, at P10, basket terminals cluster around the Purkinje AIS, similar to that seen in control Purkinje cells (Figure 4Db, compare with Figure 4Ab, arrowheads). At P20, the terminals remained scattered around the Purkinje cell base and the AIS and did not coalesce into a compact pinceau as seen in controls (Figure 4Ib, compared with Figure 4Fb, arrowhead). By P30, the basket terminals remained mislocalized at around the Purkinje soma base and the AIS (Figure 4Nb, compare with Figure 4Kb, arrowhead), indicating that loss of AnkG in Purkinje and basket cells affects pinceau organization. In *Parv-Cre*; *NFF^x/Fx^*; *AnkG^Fx/Fx^* (*Parv*; *DKO*) mice, K_v_1.2 clustering was severely disrupted at P10 (Figure 4Eb, arrowhead) as basket terminals around the Purkinje AIS were not present (compared with Figure 4Ab, arrowhead). By P20, the DKO mice showed scattered basket terminals and K_v_1.2 immunoreactivity around the soma and the AIS (Figure 4Jb, arrowhead, compare with Figure 4Fb, arrowhead, quantified in Figure 4S). By P30, K_v_1.2 staining remained diffuse and disorganized (Figure 4Ob, arrowhead, quantified in Figure 4T), with less structured pinceau formations than in either wild-type or *Pcp2-Cre*; A*nkG* or the double mutants. Notably, although some K_v_1.2 signal remained detectable, it lacked the compact bouton-like morphology characteristic of basket cell terminals that coalesce to form the pinceau around the Purkinje AIS. These results reveal that both Purkinje cell- and basket cell-derived NF186 and AnkG are required for proper pinceau assembly, and both contribute to developmental AIS organization and pinceau formation.

### Loss of NF186 and AnkG in Purkinje cells causes progressive Purkinje cell degeneration

**Figure 5:**
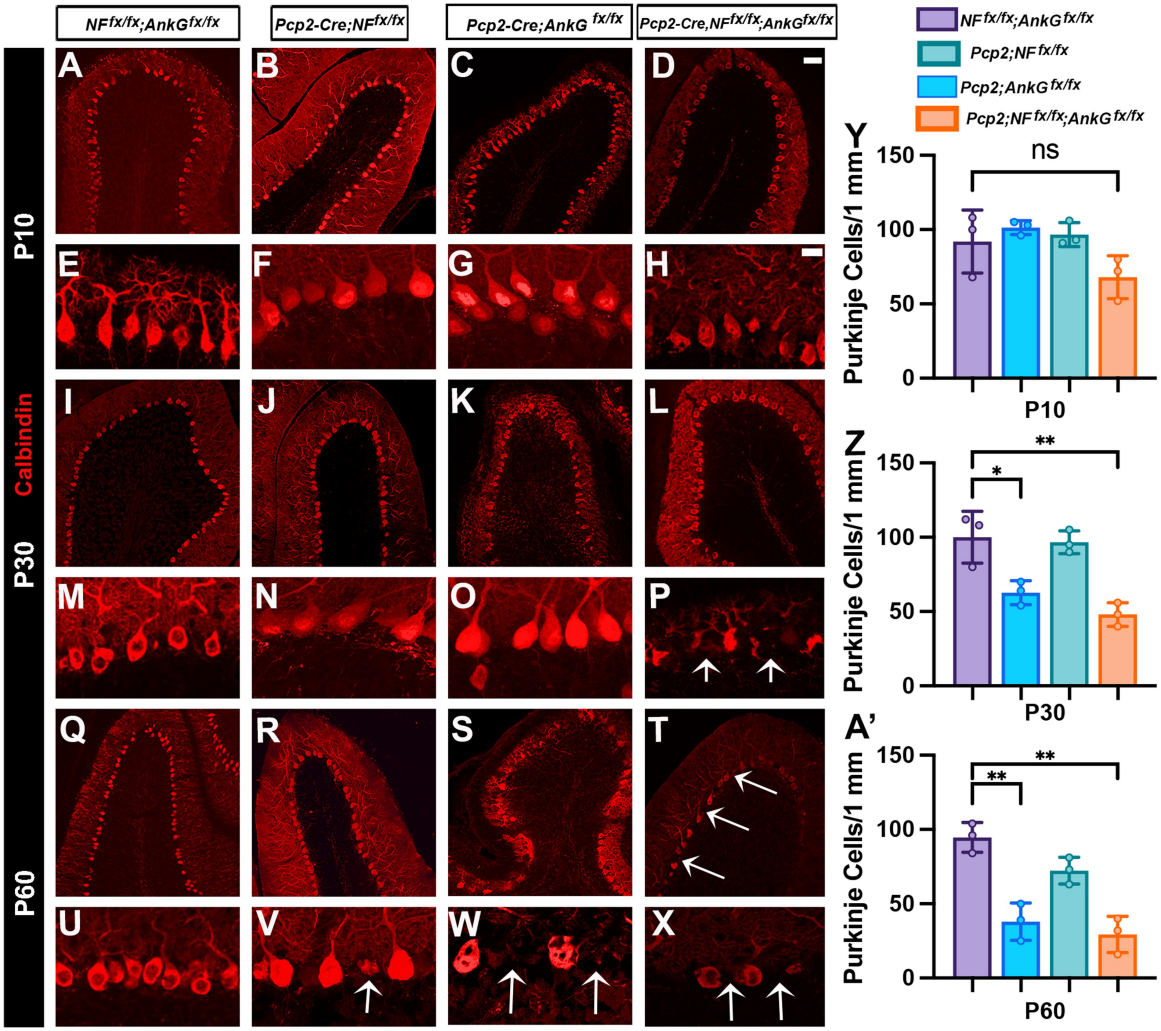
Purkinje cell-specific loss of Neurofascin and Ankyrin G leads to progressive degeneration of Purkinje cells. **(A–X)** Calbindin immunostaining in control *NF^Fx/Fx^*; A*nkG^Fx/Fx^*, *Pcp2-Cre*; *AnkG^Fx/Fx^*, *Pcp2-Cre*; *NFF^x/Fx^*, and *Pcp2-Cre*; *NFF^x/Fx^*; *AnkG^Fx/Fx^* cerebellar sections at P10 **(A–H)**, P30 **(I–P)**, and P60 **(Q–X)**. Note the morphological changes and degeneration of Purkinje cells are already visible at P10 in *Pcp2-Cre*; *NFF^x/Fx^*; *AnkG^Fx/Fx^* **(H)**. Higher magnification images show a clear loss of Purkinje cells at P30 **(P)** and P60 **(X)**. Scale bars in **A–D**, **I–L**, and **Q–T**: 100 μm. Scale bars in **E–H**, **M–P**, and **U–X**: 25 μm. Arrows point to the areas of degenerated neurons. **(Y,Z,A’)** Quantification of Purkinje cell density in cerebellar sections from all four genotypes at P30 shows a significant reduction in *Pcp2-Cre*; *NFF^x/Fx^*; *AnkG^Fx/Fx^* (10–15 images per animal were averaged for statistical analysis, *n* = 3). All data are shown as mean ± SEM; *p < 0.05, **p <0.01 or n.s. (not significant) by one-way ANOVA analysis.

Given our findings in the preceding sections showing disrupted AIS assembly, altered K_v_1.2 subcellular localization, and impaired motor performance in Purkinje cell-specific NF186/AnkG knockout mice, we wanted to investigate whether loss of NF186/AnkG could also lead to degeneration of Purkinje cell degeneration. To address this, we performed immunohistochemical analyses of cerebellar sections from control (*NF^Fx/Fx^*; *AnkG^Fx/Fx^*), Purkinje cell–specific AnkG knockout (*Pcp2-Cre*; *AnkG^Fx/Fx^*), Purkinje cell–specific NF186 knockout (*Pcp2-Cre*; *NFF^x/Fx^*), and double knockout [*Pcp2-Cre*; *NFF^x/Fx^*; *AnkG^Fx/Fx^*; (*Pcp2*; *DKO*)] mice at P10, P30, and P60 (Figures 5A–X). At P10, Purkinje cell layering and soma morphology were largely preserved across all genotypes (Figures 5A–D), and high-magnification views showed densely packed calbindin-positive Purkinje soma with intact proximal dendritic arbors (Figures 5E–H). Although *Pcp2-DKO* mice already showed a modest reduction in Purkinje cell density relative to controls (Figures 5D,H), quantification confirmed that the reduction was most apparent in the DKO group at this early stage. In the single knockouts, Purkinje cells were not significantly reduced compared to controls (Figure 5Y). By P30, Purkinje cell degeneration became statistically significant and was genotype-dependent. Control mice maintained a continuous Purkinje cell layer (Figures 5I,M), whereas *Pcp2*; *DKO* mice exhibited clear gaps and reduced Purkinje cell density (Figure 5L), with high-magnification images highlighting missing cells and disrupted local organization (Figure 5P, arrows). Notably, *AnkGKO* mice also displayed a reduction in Purkinje cell density at P30 relative to controls (Figures 5J,N), whereas *NFKO* mice appeared comparatively preserved at this stage (Figures 5K,O). These differences were reflected in quantification, with significant Purkinje cell loss in the *DKO* mice and a measurable decline in *AnkGKO* compared with controls, while *NFKO* remained closer to control levels (Figure 5Z). By P60 Purkinje cell degeneration had progressed further in *Pcp2-DKO* mice. Control animals continued to show an intact Purkinje cell layer (Figures 5Q,U), but the *Pcp2-DKO* mice displayed pronounced and widespread Purkinje cell loss, with large gaps in the Purkinje cell layer and markedly reduced calbindin-positive soma (Figures 5T,X; arrows; also see the low-magnification overview in Figure 5T). By comparison, both *AnkGKO* and *NFKO* mice showed Purkinje cell loss at P60, with the reduction more apparent in *AnkGKO* (Figures 5R,V, arrows) and detectable but generally less severe in *NFKO* (Figures 5S,W, arrow), consistent with the Purkinje cell quantification in controls (Figure 5A’). Together, these data demonstrate that Purkinje cell degeneration is progressive and is most severe following combined loss of NF186 and AnkG, while loss of AnkG alone is associated with earlier and more pronounced degeneration than loss of NF186 alone. These results support a model in which AIS integrity and its associated cytoskeletal scaffold are not only required for proper excitability and circuit function, but are also critical for long-term Purkinje neuron survival, with dual disruption of NF186 and AnkG accelerating a degenerative cascade that culminates in substantial Purkinje cell loss in the cerebellum.

### Single and dual loss of Neurofascin 186 and Ankyrin G in Purkinje cells generate distinct transcriptomic responses

**Figure 6:**
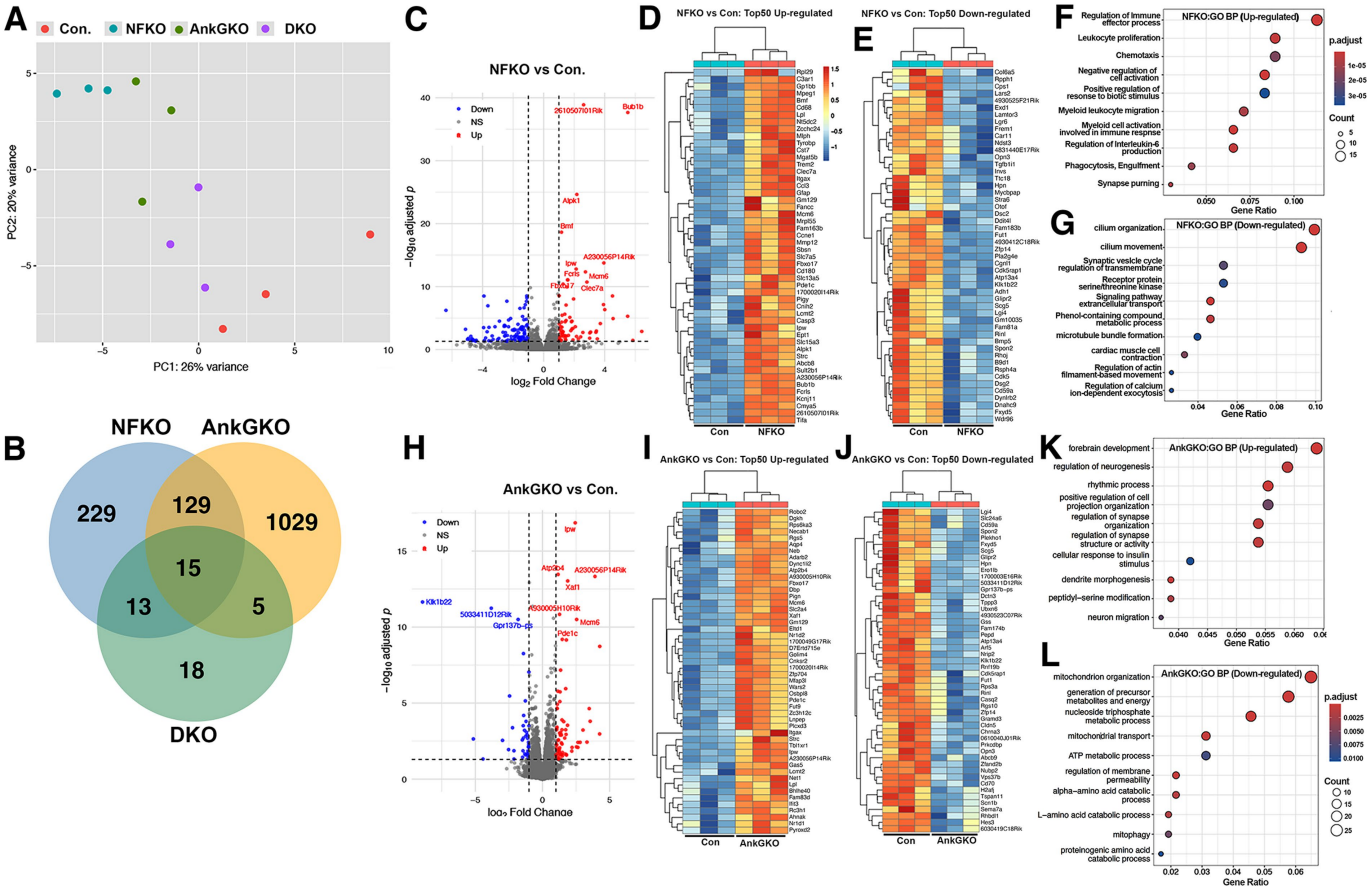
Transcriptomic profiling of *Pcp2-Cre*; *DKO* cerebellum from Neurofascin and Ankyrin G single and double mutants. **(A)** Principal component analysis (PCA) of cerebellar RNA-seq data shows tight clustering of biological replicates within each group [Control, *Pcp2-Cre*; *NFF^x/Fx^* (NF186 KO), and *Pcp2-Cre*; *AnkG^Fx/Fx^* (AnkG KO), and *Pcp2-Cre*; *NFF^x/Fx^*; *AnkG^Fx/Fx^* (DKO)], indicating strong within-group consistency and distinct transcriptomic signatures between genotypes. **(B)** Venn diagram illustrating the overlap of differentially expressed genes (DEGs) between NF186 KO vs. control, AnkG KO vs. control, and DKO vs. control comparisons, with a subset of DEGs shared by both genotypes. **(C)** Differential expression analysis of NF186 KO cerebellum versus control shown in a volcano plot, showing significantly upregulated (red) and downregulated (blue) genes (adjusted *p* < 0.05). **(D)** Heatmap of the top 50 upregulated genes in NF186 KO, highlighting enrichment for inflammatory and glial markers. **(E)** Heatmap of the top 50 downregulated genes in NF186 KO, primarily consisting of neuronal and synaptic components. **(F)** Gene Ontology (GO) enrichment analysis of upregulated genes in NF186 KO reveals activation of immune response, lipid metabolism, and gliosis-related pathways. **(G)** GO analysis of downregulated genes in NF186 KO identifies deficits in synaptic transmission, ion transport, and neuronal projection development. **(H)** Differential expression analysis of AnkG KO cerebellum versus control is shown in a volcano plot, highlighting significant up- and downregulated transcripts. **(I)** Heatmaps of the top 50 upregulated genes in AnkG KO include glial and immune-related transcripts. **(J)** Heatmaps of the top 50 downregulated genes in AnkG KO predominantly include AIS-associated and excitatory synaptic genes. **(K)** GO enrichment of upregulated genes in AnkG KO shows glial activation, nitric oxide signaling, and lipid homeostasis pathways. **(L)** GO enrichment of downregulated genes in AnkG KO includes categories related to synaptic signaling, dendrite development, and action potential generation.

To investigate the molecular changes in Purkinje cells when NF186 and AnkG are ablated either independently or jointly, we performed RNA sequencing (RNA-seq) analysis comparing cerebellum tissues from *Pcp2-Cre*; *NFF^x/Fx^*; *AnkG^Fx/Fx^* (control, Con), *Pcp2-Cre*; *NFF^x/Fx^* (NFKO), *Pcp2-Cre*; *AnkG^Fx/Fx^* (AnkGKO), and *Pcp2-Cre*; *NFF^x/Fx^*; *AnkG^Fx/Fx^* (DKO) mice together with three littermate controls (*n* = 3). Principal component analysis (PCA; Figure 6A) revealed clear clustering patterns, demonstrating distinct gene expression profiles among the genotypes. Control samples clustered tightly together, distinctly separated from NFKO, and AnkGKO samples, while DKO samples were positioned intermediate to the single KO groups and controls, suggesting complex transcriptomic changes arising from single or dual loss of NF and AnkG (Figure 6A). Venn diagram analysis showed a limited overlap among differentially expressed genes (DEGs) from all three knockout groups, indicating primarily gene-specific transcriptional regulation, with only 15 genes commonly dysregulated across all three KO conditions (Figure 6B), suggesting that loss of NF186 and AnkG elicits distinct transcriptional responses with some overlaps.

Differential gene expression analysis of NFKO versus control identified numerous significantly altered genes. Volcano plot representation highlighted a prominent set of both upregulated and downregulated genes for *NFKO* vs. control, defined by adjusted *p*-value < 0.05 and absolute fold change > 1.5 (Figure 6C). Heatmap analysis of the top 50 upregulated genes in *NFKO* vs. control showed increased expression of inflammatory and glial-associated transcripts (such as *Clec7a*, *Fcgr3*, and *Csf1r*), whereas the top 50 downregulated genes were enriched for neuronal and synaptic markers (*Snap25*, *Sv2a*, *Syp*; Figures 6D,E). Consistent with this pattern, Gene Ontology (GO) analysis of the upregulated genes identified immune response, lipid metabolism, and gliosis-related pathways, while downregulated genes were enriched for synaptic transmission, ion transport, and neuronal projection development (Figures 6F,G). These findings indicate that loss of NF186 is associated with the induction of reactive/inflammatory pathways together with the suppression of neuronal/synaptic programs.

*AnkGKO* versus control also produced a strong transcriptomic shift (Figure 6H). Heatmaps of the top altered genes showed increased expression of genes related to neuronal development and morphogenesis (*Robo2*, *Dgkh*, and *Axin2*), and reduced expression of genes involved in mitochondrial (*Atp2a2* and *Cox8b*) and metabolic function (*Got2* and *Aldh5a1*; Figures 6I,J). GO enrichment analysis supported these observations, with upregulated genes associated with neurodevelopmental and morphogenetic processes and downregulated genes associated with mitochondrial organization and amino acid catabolism (Figures 6K,L). Thus, although both single knockouts altered neuronal homeostasis, *NFKO* and *AnkGKO* showed distinct pathway-level signatures.

**Figure 7:**
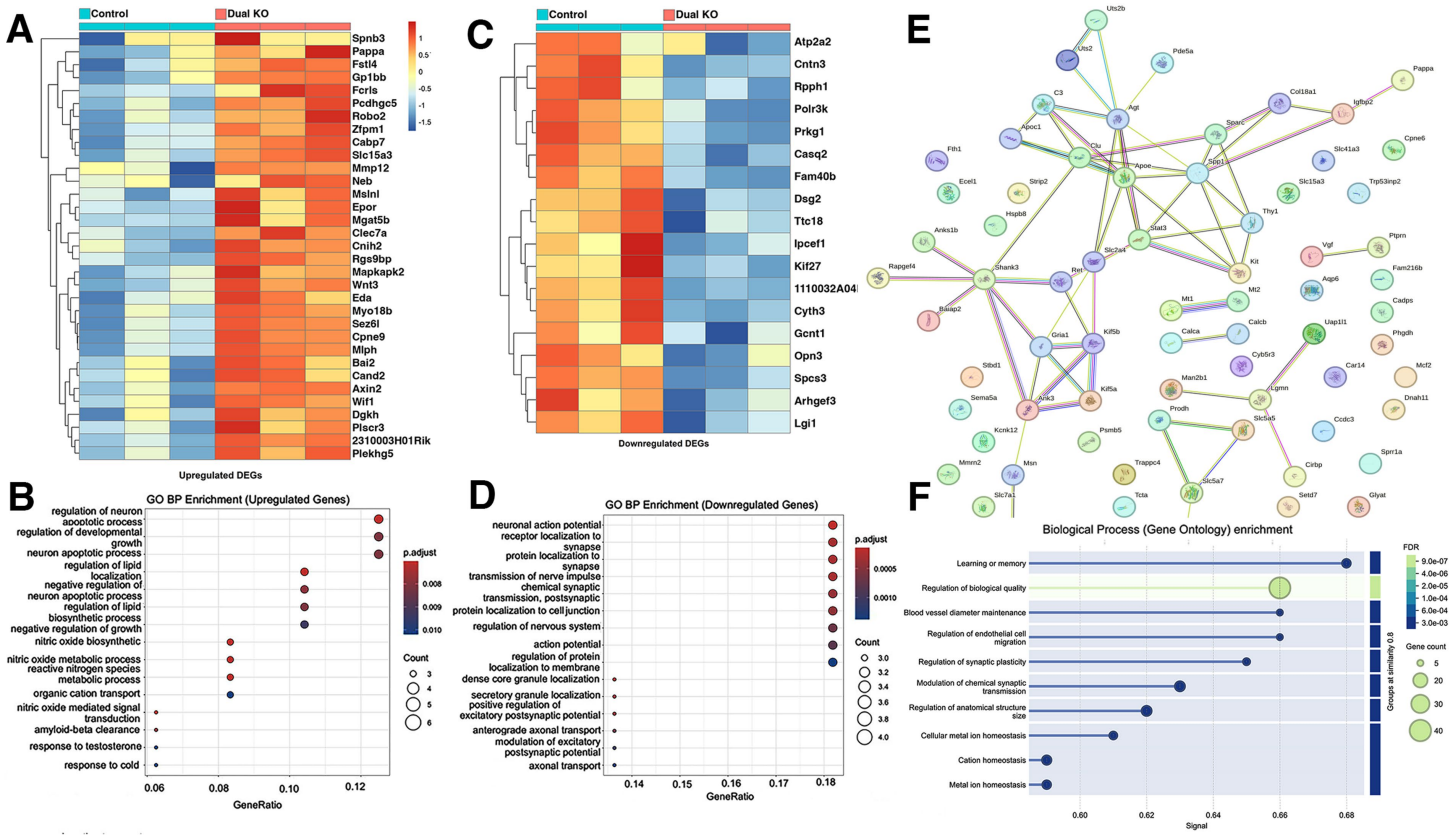
Dual loss of NF186 and Ankyrin G in Purkinje cells triggers coordinated glial activation and synaptic gene repression. **(A)** Heatmap displaying the top upregulated differentially expressed genes (DEGs) in *Pcp2-Cre*; *NFF^x/Fx^*; *AnkG^Fx/Fx^* double knockout (DKO) cerebellum compared to littermate controls *NF^Fx/Fx^*; *AnkG^Fx/Fx^*. **(B)** Gene Ontology (GO) enrichment analysis of upregulated genes reveals pathways associated with lipid metabolism, nitric oxide signaling, and reactive gliosis. **(C)** Heatmap of the top downregulated DEGs in DKO samples highlights reduced expression of neuronal and AIS-associated genes. **(D)** GO enrichment analysis of downregulated genes indicates impaired excitatory synaptic signaling, ion transport, and receptor trafficking processes. **(E)** STRING protein–protein interaction network analysis of the full DEG list (top 35 up- and downregulated genes) reveals two high-confidence clusters: a synaptic/AIS module centered around Ankyrin-G, GRIA1, and SHANK3 (red circle), and a glial/inflammatory module containing APOE, CLU, and STAT3 (blue circle). **(F)** GO enrichment analysis of the STRING network supports functional integration of neuronal and glial signaling programs, linking loss of NF186 and Ank G to downstream effects on synaptic plasticity and inflammatory signaling.

We next examined the transcriptomic consequences of the combined loss of NF186 and AnkG. In the DKO versus control comparison, heatmap analysis identified two major groups of differentially expressed genes (Figures 7A,C). Upregulated transcripts were enriched for glial and inflammatory genes, including *Apoe*, *Clu*, *Stat3*, *C3*, *Sparc*, and *Nos2* (Figure 7A). GO analysis of the upregulated genes identified lipid metabolic processes, nitric oxide signaling, and reactive gliosis pathways (Figure 7B). The downregulated transcripts included neuronal and synaptic/AIS-associated genes such as *Gria1*, *Shank3*, *Dlg4*, *Kcnk2*, and *Kif5a/b* (Figure 7C). GO analysis of the downregulated genes showed genes enriched for action potential generation, synaptic transmission, and receptor localization at the synapse (Figure 7D). STRING network analysis further resolved these DEGs into two high-confidence modules: a neuronal/synaptic cluster centered on *AnkG*, *GRIA1*, *SHANK3*, and *DLG4* (red circle) and a glial-immune cluster centered on *APOE*, *CLU*, *STAT3*, and *C3* (blue circle; Figures 7E,F). Together, these data indicate that combined AIS disruption by loss of NF186 and AnkG is associated with repression of neuronal/synaptic programs and a prominent glial-inflammatory response.

**Figure 8:**
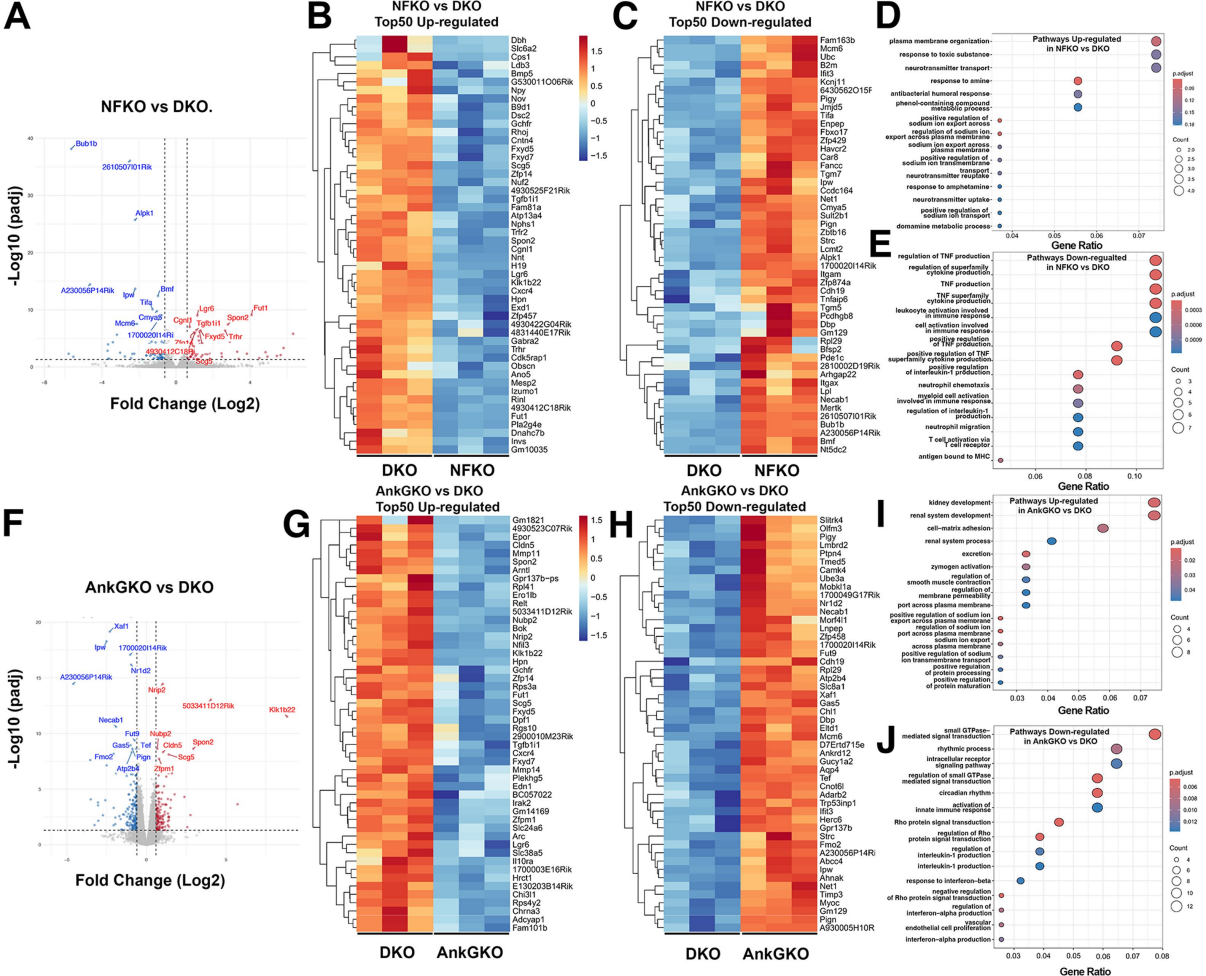
Direct transcriptomic comparison of Neurofascin and Ankyrin G single versus double mutant cerebella reveals changes in distinct genetic pathways. **(A)** Differential expression analysis of *Pcp2-Cre*; *NFF^x/Fx^* (NFKO) cerebellum versus *Pcp2-Cre*; *NFF^x/Fx^*; *AnkG^Fx/Fx^* double knockout (DKO) shown in a volcano plot showing significantly upregulated (red) and downregulated (blue) genes (adjusted *p* < 0.05). **(B)** Heatmap of the top 50 upregulated genes in NFKO vs. DKO (genes higher in DKO relative to NFKO). **(C)** Heatmap of the top 50 downregulated genes in NFKO vs. DKO (genes lower in relative DKO to NFKO). **(D)** Gene ontology (GO) enrichment analysis of genes upregulated in NFKO vs. DKO identifies pathways including plasma membrane organization, neurotransmitter transport/uptake, and sodium ion transport–related processes. **(E)** GO enrichment analysis of genes downregulated in NFKO vs. DKO identifies immune-related pathways, including TNF production/regulation, cytokine production, leukocyte activation, neutrophil chemotaxis/migration, and antigen presentation (MHC). **(F)** Differential expression analysis of *Pcp2-Cre*; *AnkG^Fx/Fx^* (AnkGKO) versus DKO cerebellum shown as a volcano plot highlighting significantly upregulated (red) and downregulated (blue) genes (adjusted *p* < 0.05). **(G)** Heatmap of the top 50 upregulated genes in AnkGKO vs. DKO (genes higher in DKO relative to AnkGKO). **(H)** Heatmap of the top 50 downregulated genes in AnkGKO vs. DKO (genes lower in DKO relative to AnkGKO). **(I)** GO enrichment analysis of genes upregulated in AnkGKO vs. DKO identifies pathways including kidney/renal system development, cell–matrix adhesion, and membrane permeability/transport (including sodium ion transport and export across the plasma membrane). **(J)** GO enrichment analysis of genes downregulated in AnkGKO vs. DKO highlights signaling and immune-related pathways, including small GTPase–mediated signal transduction, rhythmic/circadian processes, Rho protein signal transduction, and interferon/innate immune response categories (e.g., interleukin-1 production, response to interferon-β, and interferon-*α* production).

To determine how each NF186 and AnkG single-knockout states relate to the DKO transcriptome, we next performed direct *NFKO* versus DKO and *AnkGKO* versus DKO comparisons (Figure 8). In the *NFKO* versus DKO comparison, volcano plot and heatmap analyses showed clear separation between the two genotypes (Figures 8A–C). Several neuronal/Purkinje-associated transcripts, including *Car8*, *Necab1*, and *Chl1*, were relatively higher in *NFKO* than in DKO, whereas DKO showed relatively increased expression of reactive/injury-associated genes such as *Cxcr4* (Figures 8B,C). Consistent with these gene-level differences, genes increased in *NFKO* relative to DKO were enriched for membrane and transport-related pathways, whereas genes increased in DKO relative to *NFKO* were enriched for immune-related processes, including leukocyte and neutrophil-associated pathways (Figures 8D,E). A similar pattern emerged in the *AnkGKO* versus DKO comparison. Volcano plot and heatmap analyses again demonstrated clear genotype separation (Figures 8F–H). *AnkGKO* samples showed relatively higher expression of neuronal signaling and excitability-related genes, including *Camk4*, *Ube3a*, *Ptpn4*, *Adarb2*, *Slc8a1*, and *Atp2b4*, whereas DKO samples showed relatively higher expression of reactive/immune- and barrier-associated genes such as *Chi3l1*, *Il10ra*, and *Cldn5* (Figures 8G,H). GO analysis showed that genes increased in *AnkGKO* relative to DKO were enriched for developmental/adhesion and ion transport-related processes, whereas genes increased in DKO relative to *AnkGKO* were enriched for GPCR signaling and innate immune/interferon-associated pathways (Figures 8I,J). To independently validate the RNA-seq dataset, we performed qRT-PCR analysis of selected representative transcripts in cerebellar RNA from control, *NFKO*, *AnkGKO*, and DKO mice. The qRT-PCR results were broadly consistent with the RNA-seq data, confirming upregulation of reactive/inflammatory genes (*Apoe*, *Clu*, *Stat3*, *C3*, and *Cxcr4*) and downregulation of neuronal/Purkinje-associated genes (*Car8*, *Snap25*, *Syp*, *Gria1*, and *Adarb2*) across mutant genotypes, with the strongest changes typically observed in DKO samples (Supplementary Figure 1; Supplementary Table 1). Taken together, these transcriptomic analyses show that loss of NF186 or AnkG causes distinct molecular changes in gene expression with some overlap, while DKO is characterized by a stronger neuroinflammatory/reactive signature together with broader reduction of neuronal/Purkinje-associated gene expression. These findings further indicate that combined disruption of NF186 and AnkG drives Purkinje cells into a transcriptional state that is distinct from either single mutant alone.

## Discussion

This study set out to determine how the AIS membrane protein NF186 and the AIS cytoskeletal scaffolding protein AnkG contribute to Purkinje neuron structural and functional integrity when they are ablated selectively and simultaneously in Purkinje cells. Consistent with this rationale, the Purkinje-restricted loss of these two master organizers destabilized the AIS and produced broad cellular, molecular, and cerebellar circuit consequences. We observed that Purkinje neurons lacking NF186 and AnkG failed to maintain a coherent AIS structure, showed disrupted localization of key ion channels, and progressively underwent neurodegeneration. These structural alterations were accompanied by progressive Purkinje cell degeneration and impaired motor performance. Finally, transcriptomic profiling revealed that NF186 and AnkG deletion elicited partially distinct gene-expression programs, and that combined loss produced a transcriptional state distinguishable from either single knockout. Together, our findings underscore the critical role of AIS-specific adhesion and cytoskeletal complexes in sustaining Purkinje neuronal function and cerebellar motor control.

### Neurofascin and Ankyrin G in the organization and maintenance of AIS proteins

The ablation of NF186 and AnkG essentially abolished the Purkinje AIS scaffold. βIVSpec, normally enriched at the AIS cytoskeletal lattice, was severely disrupted, indicating collapse of the intracellular scaffold that links AnkG to the actin cytoskeleton. This is in line with established models of AIS assembly: AnkG is required to maintain NF186 and βIVSpec at the AIS (Ango et al., 2004), and NF186 also helps to stabilize AnkG and βIVSpec through extracellular glial protein interactions (Buttermore et al., 2012). Previous studies demonstrated that Purkinje neurons lacking NF186 failed to establish a mature and normal AIS (Buttermore et al., 2012). Consistent with these prior observations, our Purkinje cell–specific single-knockout analyses revealed distinct temporal requirements for NF186 and AnkG in maintaining AIS βIVSpec enrichment.

In *NFKO* mice, βIVSpec remained detectable at the developing AIS at P10, but its presence at the AIS progressively declined by P20 and was nearly absent by P30, indicating that NF186 is particularly important for stabilizing βIVSpec during AIS maturation rather than for its initial recruitment. The rapid loss of βIVSpec also aligns with the idea that AnkG is required to anchor the Spectrin/actin cytoskeleton at the AIS. Without AnkG, βIVSpec fails to remain clustered at the axon’s proximal segment and instead disperses more rapidly. Indeed, in Purkinje cell–specific *AnkGKO* mice, βIVSpec clustering at the AIS was already markedly reduced at P10 and remained weak thereafter, closely resembling the double-knockout phenotype and supporting an essential role for AnkG in early AIS scaffold assembly. Interestingly, our results from *NF/AnkG* double mutants suggest that there may be a brief window early in development (around P10) when some βIVSpec is able to localize to the AIS that is still under development, even in the absence of NF186/AnkG, potentially via other scaffolding interactions. However, such partial assembly is not sustained. Importantly, the combined loss of NF186 and AnkG produced the most severe and persistent disruption of βIVSpec enrichment, exceeding the progressive decline observed in *NFKO* and matching the early, profound deficit seen in *AnkGKO*. Together, these genotype comparisons support a model in which AnkG is required for robust early recruitment/anchoring of the AIS Spectrin–Actin lattice, whereas NF186 contributes prominently to the stabilization and long-term maintenance of this scaffold during postnatal maturation. In Purkinje-specific NF186 KO, AnkG persists at the AIS (Buttermore et al., 2012), and in the current study, βIV Spectrin also remains detectable during the early postnatal period in NF186 KO, but is undetectable after P30. Our current single-KO dataset extends this conclusion by defining the timing of βIVSpec loss in *NFKO* (present at P10, reduced at P20, and nearly absent by P30), while demonstrating that *AnkGKO* exhibits an earlier and more severe defect in AIS βIVSpec clustering beginning at P10. In contrast, double NF186/AnkG deletion leads to rapid βIVSpec loss, underscoring the essential role of these proteins in stabilizing the AIS cytoskeleton. This progressive βIVSpec loss suggests ongoing AIS disassembly, which could have downstream effects on the localization of ion channels and the excitability of these neurons. Consistent with this interpretation, disruption of βIVSpec was accompanied by progressive failure of sodium channel clustering at the AIS, with *AnkGKO* and DKO showing very weak AIS Na_v_ immunoreactivity already at P10 and *NFKO* showing a delayed decline that became pronounced by P20–P30. Interestingly, we observed that βIVSpec redistributes to the Purkinje cell membrane in the absence of NF186/AnkG in later stages, as seen at P60. Thus, removal of both NF186 and its cytoskeletal anchor AnkG prevents the AIS structure altogether, revealing no apparent compensatory mechanisms when the two core components are simultaneously lost, and βIVSpec now localizes to the Purkinje cell membrane, potentially interacting with other membrane or cytoskeletal proteins. The appearance of βIVSpec at non-AIS membrane compartments at later stages suggests that, when AIS anchoring fails, βIVSpec may engage alternative interactions elsewhere in the Purkinje cell membrane, but these are insufficient to reconstitute an AIS-like scaffold.

### Clustering of sodium channels at the Purkinje axon initial segment and organization of the basket cell pinceau

A major functional consequence of AIS scaffold loss was the mislocalization of voltage-gated ion channels that normally concentrate at/or around the Purkinje AIS. In control Purkinje cells, high-density Na_v_ channels (including Na_v_1.6) at the AIS trigger spontaneous action potential firing (Buttermore et al., 2012). In NF186-deficient Purkinje neurons, spontaneous firing is abolished, highlighting that proper Na_v_ clustering at the AIS is required for Purkinje excitability (Buttermore et al., 2012). Consistent with this framework, our data indicate that loss of core AIS organizers compromises the ability of Purkinje neurons to maintain sodium channel enrichment at the proximal axon. Importantly, the single-knockout comparisons refine the interpretation: AnkG loss produced an early and pronounced deficit in AIS Na_v_ channel enrichment, whereas NF186 loss permitted initial Na_v_ accumulation during early development but was associated with progressive loss of AIS Na_v_ localization during maturation. Together, these patterns support a model in which AnkG-dependent scaffolding is critical for robust early organization of the Na_v_-rich AIS domain, while NF186 contributes to long-term stabilization of channel enrichment as the AIS matures. Although direct electrophysiological measurements were not performed here, the magnitude and timing of Na_v_ disorganization observed in the *AnkGKO* and *NF/AnkG* double knockout genotypes would be expected to reduce Purkinje output and compromise cerebellar circuit function.

We also observed disruptions in the organization of K_v_ channel–rich basket terminals that form the pinceau around the Purkinje AIS. Under normal conditions, K_v_1.1/1.2 channels are concentrated in basket cell axon terminals at the pinceau, where they shape terminal excitability and contribute to fast ephaptic inhibition of Purkinje neurons (Xie et al., 2010; Buttermore et al., 2012). Our results are consistent with the idea that maintaining a stable postsynaptic AIS domain is important for proper spatial confinement of these presynaptic specializations. In Purkinje-targeted mutants, the redistribution of the K_v_1.2 signal along the AIS region suggests that when the AIS scaffold is destabilized, basket terminals may still contact the Purkinje proximal axon but fail to maintain the sharply confined, mature pinceau organization. Moreover, the Parv-Cre mutant analyses support a parallel requirement within the basket cell lineage, consistent with prior work showing that coordinated NF186-dependent mechanisms in pre- and postsynaptic compartments contribute to pinceau maturation (Buttermore et al., 2012). Taken together, these findings support the view that AIS scaffolds influence the precise distribution of ion channels on both sides of the basket–Purkinje interface, thereby helping to align Purkinje excitability with timely inhibitory control.

The loss of NF186 and AnkG also impacted the morphological integrity of the basket cell pinceau in a manner consistent with impaired stabilization rather than a simple failure of initial targeting. In control mice, an AIS-associated NF186 gradient and associated scaffolding mechanisms contribute to basket axon collateral targeting to the AIS (Ango et al., 2004), but subsequent work has emphasized that NF186 alone is not sufficient to attract basket axons and that presynaptic programs, including neuropilin-1–dependent mechanisms, play critical roles in subcellular targeting (Telley et al., 2016). Our data, therefore, fit best with a cooperative model in which postsynaptic AIS integrity provides stabilizing and organizational cues that act together with presynaptic guidance and terminal differentiation programs. When these cues are weakened -either by disrupting postsynaptic AIS components in Purkinje cells or by perturbing basket cell–intrinsic mechanisms -basket terminals may form but fail to consolidate into the compact, properly positioned pinceau. Such abnormalities would be expected to reduce the precision of perisomatic inhibition and perturb the excitation/inhibition balance in the cerebellar cortex, contributing to Purkinje dysfunction and the emergence of motor coordination deficits.

### Purkinje cell axon initial segment disorganization and cerebellar ataxia

A striking outcome of Purkinje-specific AnkG/NF186 loss was progressive neurodegeneration accompanied by motor coordination deficits. Over time, affected Purkinje cells showed signs of axonal pathology and ultimately cell death in 50% of Purkinje cells by P60, which manifested functionally as cerebellar ataxia. This finding reveals that beyond acute electrophysiological impairment, AIS loss can initiate degenerative processes. One explanation is that AIS disruption removes critical support for neuronal polarity and axonal transport, making Purkinje axons susceptible to adverse physiological changes leading to their degeneration. Indeed, the AIS has been proposed to act as a “shield” for the axon, and its breakdown is often a harbinger of axonopathy in several disease models (Watanabe et al., 2012). Our data align with earlier reports that chronic disorganization of the Purkinje AIS or nodes of Ranvier leads to axonal degeneration and neurological deficits (Watanabe et al., 2012; Satake et al., 2017).

The single-knockout comparisons further refine this interpretation. While the double knockout mice displayed the most severe Purkinje cell degeneration, AnkG loss alone was associated with earlier and more pronounced structural deficits at the AIS and showed a stronger trend toward later functional impairment and Purkinje cell loss than NF186 loss alone. This pattern is consistent with a model in which AnkG-dependent cytoskeletal scaffolding is especially important for establishing and maintaining the core AIS framework, whereas NF186 contributes prominently to stabilization of AIS organization over time, with the combined loss accelerating failure of both scaffolding and adhesive support mechanisms. Our findings extend prior work showing that perturbing NF186-dependent mechanisms across Purkinje and basket cells can drive Purkinje neuron degeneration and severe ataxia (Buttermore et al., 2012), and complement studies demonstrating that loss of other axonal scaffolding proteins (e.g., Band 4.1B and Whirlin) can induce axonal pathology and motor dysfunction (Saifetiarova and Bhat, 2019). Together, these studies support the broader idea that integrity of specialized axonal domains is closely linked to long-term Purkinje neuron survival and cerebellar function and raise the possibility that AIS maintenance defects could contribute to certain forms of cerebellar neurodegeneration.

A recent study showed that AnkG expressed in oligodendrocytes plays a critical role in maintaining paranodal junction integrity and proper axoglial interactions, particularly during aging (Ding et al., 2024). Conditional deletion of AnkG in glial cells led to progressive deterioration of node-paranode architecture, impaired saltatory conduction, and behavioral deficits reminiscent of neuropsychiatric conditions. These findings extend the importance of AnkG beyond neurons and into glial function, highlighting its role in long-term axonal health and circuit stability. Our study complements and extends these findings by focusing on Purkinje neuron-specific deletion of AnkG and NF186 and demonstrates that loss of AnkG and NF186 in neurons themselves leads to rapid AIS disorganization, mislocalization of ion channels, pinceau synapse disruption, and ultimately neurodegeneration. Moreover, our transcriptomic profiling of double knockouts reveals upregulation of injury and stress-response pathways and downregulation of synaptic genes, suggesting that neuronal AnkG is not only critical for AIS structure but also regulates long-term neuronal health at the transcriptional level. Furthermore, AnkG single knockout shares major features with the double knockout, including progressive AIS destabilization and a substantially more severe phenotype than *NFKO* alone. This difference likely reflects the distinct hierarchical roles of AnkG and NF186 in maintaining the Purkinje cell AIS during postnatal maturation. In *NFKO* mice, AnkG remains localized at the AIS, indicating that the core AnkG-based scaffold can still be retained despite loss of NF186. In contrast, AnkG ablation causes a progressive postnatal loss of NF186, suggesting that NF186 stability at the AIS depends on an AnkG-based cytoskeletal scaffold that is essential for maintaining NF186 at the AIS. Thus, AnkG deletion effectively compromises both AnkG-dependent scaffolding and the continued retention of NF186, thereby producing a phenotype that more closely resembles the double knockout. These findings support the idea that AnkG occupies a more upstream organizational role in AIS maintenance, whereas NF186 creates an adhesive structure with other extracellular molecules between neurons and other cell types, and further support a model in which AnkG acts as a central hub in maintaining axonal integrity, circuit functionality, and neuronal health throughout life.

### Loss of Neurofascin and Ankyrin G causes Purkinje cell transcriptomic changes

To further probe the mechanisms linking loss of NF186 and AnkG to Purkinje cell degeneration, we analyzed the transcriptomic signatures from Purkinje cell–targeted *NFKO*, *AnkGKO*, and double knockout mice. Notably, these datasets revealed coordinated changes consistent with neuronal stress and altered cellular homeostasis together with reduced expression of many Purkinje-enriched neuronal/synaptic transcripts, a pattern compatible with an injury-response state that emerges as AIS organization fails. Rather than implying a single causal sequence, we interpret these changes as reflecting a convergence of impaired axonal domain organization, altered excitability, and disrupted connectivity that collectively shift Purkinje cells toward a stressed and functionally compromised state. In line with this view, disruption of AIS anchoring mechanisms can be expected to impair ion channel compartmentalization and action potential initiation, and prior work has linked AIS disassembly to ion channel mislocalization, reduced excitability, and structural instability (Hedstrom et al., 2008; Saifetiarova et al., 2017). Reactive gliosis is also commonly associated with neuronal stress in these settings, suggesting the emergence of neuron–glia crosstalk as neuronal degeneration progresses.

A key insight from the single-knockout analysis is that NF186 loss and AnkG loss are not transcriptionally redundant. Every single knockout showed a partially distinct signature, consistent with their different positions in the AIS molecular hierarchy, and the double knockout exhibited a transcriptional state distinguishable from either single mutant. In particular, relative to the single knockouts, the double knockout was characterized by a stronger enrichment of inflammatory/reactive programs, consistent with robust induction of glial-associated genes and inflammatory signaling modules. This pattern is consistent with the idea that more severe and persistent AIS disruption—when both adhesive (NF186-dependent) and cytoskeletal (AnkG-dependent) layers are compromised—amplifies secondary stress signaling and non-cell-autonomous glial responses, rather than simply increasing the magnitude of a single-KO program. At the same time, the single-KO datasets suggest that subsets of neuronal/Purkinje identity and signaling transcripts are more preserved in every single mutant than in the double knockout, reinforcing the view that combined loss of NF186/AnkG in Purkinje cells may influence functions that are not limited to AIS disorganization alone but are associated with broader cellular pathways and programs within Purkinje cells.

We also observed suppression of genes linked to synaptic function and neurotransmission, consistent with reduced functional output and altered circuit engagement in AIS-disrupted Purkinje neurons. In parallel, multiple categories related to cellular stress and remodeling were elevated, including pathways associated with lipid handling and complement-linked inflammatory signaling, which have been implicated in neurodegenerative contexts. Importantly, these signatures should not be interpreted as proving that AIS disruption alone is sufficient to trigger cell death; rather, they suggest that AIS instability is coupled to broader changes in neuronal state that may increase vulnerability over time. In this regard, parallels can be drawn with studies showing that perturbations of upstream signaling pathways can converge on AIS stability. For example, Tsc1 deletion in Purkinje neurons causes mTOR pathway dysregulation and has been associated with altered AIS organization and reduced AnkG clustering (Brown et al., 2025), suggesting that growth and stress signaling can influence AIS maintenance. Conversely, AIS disruption itself may engage retrograde stress pathways—including altered calcium handling, oxidative stress, and trafficking defects—that further amplify vulnerability and remodeling. Consistent with this possibility, we observed changes in gene sets linked to ion homeostasis and signaling regulation, which may reflect compensatory responses to altered excitability or more global cytoskeletal stress. Together, our molecular profiling supports the emerging view that NF186 and AnkG are associated with distinct but interacting programs that shape Purkinje cell state during development and disease-relevant stress. Rather than “synergistic” in the strict statistical sense, our data support a complementary model in which loss of each AIS component produces partially unique transcriptional consequences in the Purkinje cells, while combined loss is associated with a more pronounced shift toward reactive/inflammatory signaling and a broader repression of neuronal/synaptic gene modules. These transcriptional alterations provide a molecular framework for understanding how AIS destabilization can be linked to progressive Purkinje dysfunction and degeneration.

In summary, our Purkinje cell-specific knockout mice reveal that central NF186 and AnkG are essential for the formation, maturation, and long-term stabilization of the AIS, as well as for Purkinje cell function and survival. The AIS serves as an organizing center that coordinates electrical excitability through Na_v_/K_v_ channel clustering, synaptic integration through maintaining pinceau architecture, and structural integrity through axonal cytoskeletal anchoring, such as by βIV Spectrin. When this highly specialized domain is destabilized—most prominently in NF186 and AnkG double knockout—Purkinje cells lose their autonomous pacemaking ability, their synaptic input–output organization is perturbed, and ultimately, they succumb to degeneration. Importantly, the single-knockout comparisons indicate that AnkG and NF186 contribute in partially distinct ways, with AnkG playing a dominant role in establishing and maintaining the AIS cytoskeletal scaffold and NF186 contributing prominently to stabilization of AIS organization during maturation; combined loss produces the most severe and persistent disruption. From a broader perspective, our work emphasizes that intact AIS function is essential for cerebellar circuit stability and motor control (Adler, 2023; Jenkins and Bender, 2025) and supports a mechanistic link between AIS disruption and cerebellar ataxia-like phenotypes. These findings raise the possibility that strategies aimed at preserving AIS integrity—either by maintaining key AIS scaffolding/adhesion components or by limiting AIS disassembly during stress—could help sustain neuronal firing capacity and reduce vulnerability to degeneration. By ensuring the retention of critical AIS proteins or preventing their disassembly, we may preserve neuronal firing competency and forestall degeneration. In conclusion, the present study highlights the AIS as a key subcellular area underpinning Purkinje neuron health and disease, and it broadens our understanding of how neuronal circuits depend on AIS stability and integrity for lifelong function.

## Data Availability

The RNA sequencing datasets generated in this study are publicly available in the NCBI Gene Expression Omnibus (GEO) under accession number GSE322737 (https://www.ncbi.nlm.nih.gov/geo/query/acc.cgi?acc=GSE322737). All other data supporting the conclusions of this article, including raw imaging data and quantitative datasets, are available from the corresponding author upon a formal request.
