# Supplementary material for: Purkinje cell-specific loss of Neurofascin and Ankyrin G causes disruption of axon initial segments, neurodegeneration, and cerebellar ataxia
Source: Front Cell Neurosci. 2026 Apr 13;20:1690466. doi: 10.3389/fncel.2026.1690466 (PMC13111106; doi:10.3389/fncel.2026.1690466)
Supplement: Supplementary file 1 [file Data_Sheet_1.pdf]

Supplementary Figure 1. qRT-PCR validation of representative RNA-seq changes in cerebellar tissue from control, NF-KO, AnkG-KO, and DKO mice.

qRT-PCR analysis confirmed expression changes in selected genes identified by RNA-seq, including reactive/inflammatory genes Apoe (A), Clu (B), Stat3 (C), C3 (D), and Cxcr4 (E), as well as neuronal/Purkinje-associated genes Car8 (F), Snap25 (G), Syp (H), Gria1 (I), and Adarb2 (J). Expression levels were normalized to internal reference gene (*Actb*) and plotted relative to control. Data are presented as mean ± SEM. Statistical significance was determined by one-way ANOVA followed by multiple-comparisons testing. *p < 0.05, **p < 0.01, ***p < 0.001, **** p < 0.0001.

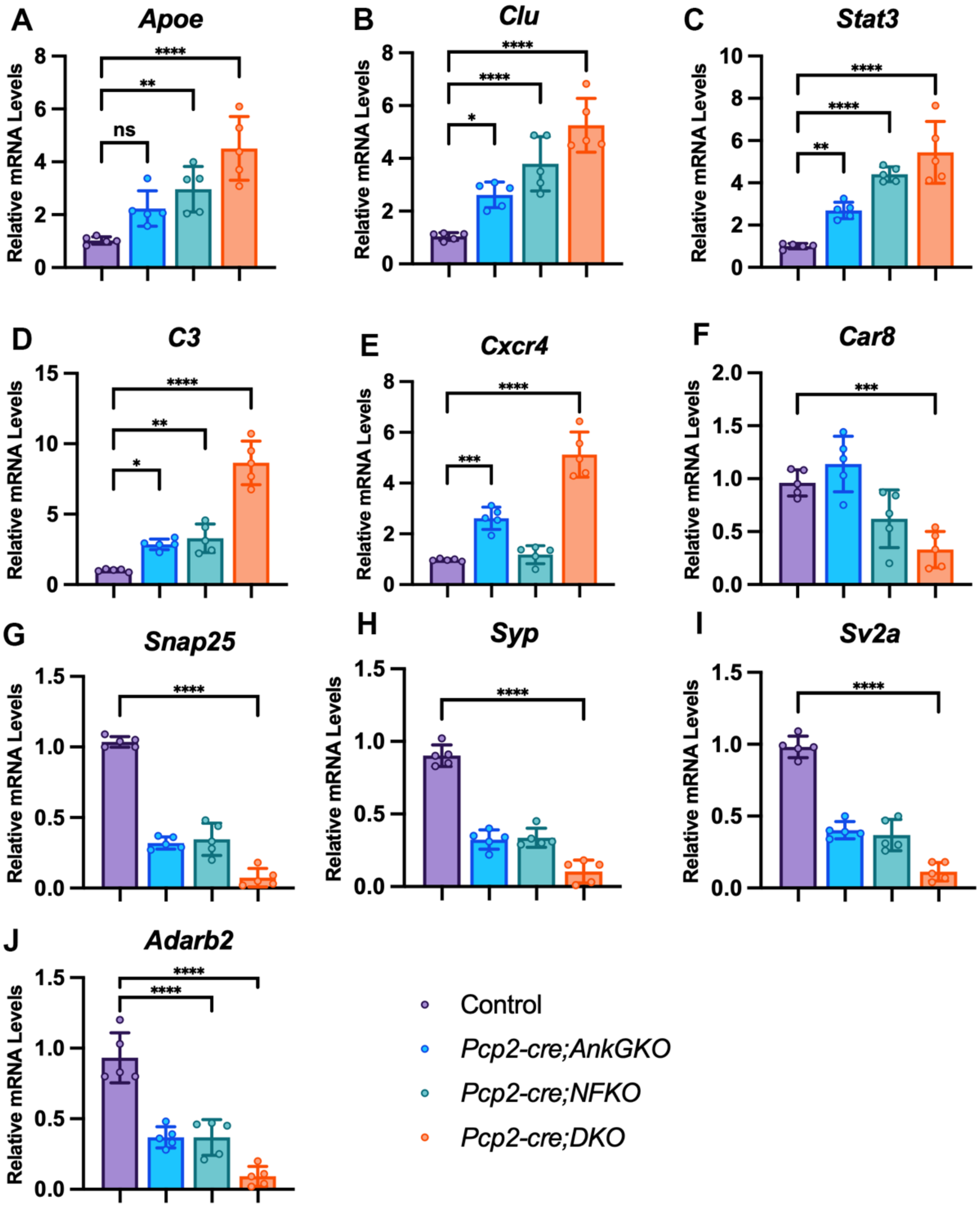

Supplementary Table 1: Primers for RT-PCR validation

Gene Name	GenBank Accession#	Primers
Apoe	NM_009696	For: *CTGACAGGATGCCTAGCCG*<br>Rev: *CGCAGGTAATCCCAGAAGC*
Clu	NM_013492	For: *AGCAGGAGGTCTCTGACAATG*<br>Rev: *GGCTTCCTCTAAACTGTTGAGC*
Stat3	NM_011486	For: *CAATACCATTGACCTGCCGAT*<br>Rev: *GAGCGACTCAAACTGCCCT*
C3	NM_009778	For: *CCAGCTCCCCATTAGCTCTG*<br>Rev: *GCACTTGCCTCTTTAGGAAGTC*
Cxcr4	NM_009911	For: *GAAGTGGGGTCTGGAGACTAT*<br>Rev: *TTGCCGACTATGCCAGTCAAG*
Car8	NM_007592	For: *ATGGCTGACCTGAGCTTCATT*<br>Rev: *ACCTTCCTCGTAACCCCACT*
Snap25	NM_011428	For: *CAACTGGAACGCATTGAGGAA*<br>Rev: *GGCCACTACTCCATCCTGATTAT*
Syp	NM_009305	For: *CAGTTCCGGGTGGTCAAGG*<br>Rev: *ACTCTCCGTCTTGTTGGCAC*
Sv2a	NM_022030	For: *GGCTTTCGAGACCGAGCAG*<br>Rev: *GACCTTCGGGAATACTCATCCT*
Adarb2	NM_052977	For: *GTCTGGAGGGCTAAGCAGTC*<br>Rev: *GCAAGGAAGGTTGACAGTATGC*
Actb	NM_007393	For: *GGCTGTATTCCCCTCCATCG*<br>Rev: *CCAGTTGGTAACAATGCCATGT*
